# Dynamic multi-site phosphorylation by Fyn and Abl drives the interaction between CRKL and the novel scaffolding receptors DCBLD1 and DCBLD2

**DOI:** 10.1042/BCJ20170615

**Published:** 2017-11-21

**Authors:** Anna M. Schmoker, Jaye L. Weinert, Kyle J. Kellett, Hannah E. Johnson, Ryan M. Joy, Marion E. Weir, Alicia M. Ebert, Bryan A. Ballif

**Affiliations:** Department of Biology, University of Vermont, 109 Carrigan Drive, 120A Marsh Life Sciences, Burlington, VT, 05405, USA

## Abstract

Discoidin, CUB, and LCCL Domain-containing (DCBLD) 2 is a neuropilin-like transmembrane scaffolding receptor with known and anticipated roles in vascular remodeling and neuronal positioning. DCBLD2 is also upregulated in several cancers and can drive glioblastomas downstream of activated Epidermal Growth Factor Receptor. While a few studies have shown either a positive or negative role for DCBLD2 in regulating growth factor receptor signaling, little is known about the conserved signaling features of DCBLD family members that drive their molecular activities. We previously identified DCBLD2 tyrosine phosphorylation sites in intracellular YxxP motifs that are required for the phosphorylation-dependent binding of the signaling adaptors CRK and CRKL (CT10 regulator of kinase and CRK-Like). These intracellular YxxP motifs are highly conserved across vertebrates and between DCBLD family members. Here, we demonstrate that, as for DCBLD2, DCBLD1 YxxP motifs are required for CRKL-SH2 binding. We report Src family kinases (SFKs) and Abl differentially promote the interaction between the CRKL-SH2 domain and DCBLD1 and DCBLD2, and while SFKs and Abl each promotes DCBLD1 and DCBLD2 binding to the CRKL-SH2 domain, the effect of Abl is more pronounced for DCBLD1. Using high performance liquid chromatography coupled with tandem mass spectrometry, we quantified phosphorylation at several YxxP sites in DCBLD1 and DCBLD2, mapping site-specific preferences for SFKs and Abl. Together these data provide a platform to decipher the signaling mechanisms by which these novel receptors drive their biological activities.

## INTRODUCTION

Proper neurodevelopment requires precise temporal and spatial regulation of a complex array of signaling molecules, the regulation of which remains largely uncharacterized. CT10 regulator of kinase (CRK) and CRK-like (CRKL) are ubiquitously expressed intracellular signaling adaptors critical to neuronal positioning in the embryonic brain, as well as to many fundamental cellular processes such as proliferation, differentiation, apoptosis, and focal adhesion dynamics [1–5]. CRK and CRKL each possess a single Src homology 2 (SH2) domain that binds to phosphorylated tyrosine residues in YxxP motifs, linking signaling molecules harboring such motifs with downstream effectors bound to the CRK and CRKL SH3 domains.

Previously, we reported a proteomics screen for novel Src Family Kinase (SFK) substrates that, when phosphorylated, would bind to the CRKL SH2 domain [6]. This screen identified the transmembrane protein Discoidin, CUB, and LCCL domain-containing 2 (DCBLD2; also endothelial and smooth muscle cell-derived neuropilin-like, ESDN) as a novel phosphotyrosine-dependent CRKL-SH2 binding partner. DCBLD2 possesses a similar ectodomain structure to that of neuropilins, critical co-receptors for guidance cues in neuronal pathfinding. The seven YxxP motifs residing within the intracellular sequence of DCBLD2 are highly conserved among vertebrates, and are essential for the phosphorylation-dependent binding of DCBLD2 to the CRKL SH2 domain. Furthermore, it was determined that SFKs were sufficient for, but not the only tyrosine kinases capable of, inducing the interaction of DCBLD2 with CRKL. Our initial study also identified four specific sites on DCBLD2 of regulated tyrosine phosphorylation, three of which were in YxxP motifs [6].

DCBLD2 has been reported to be involved in vasculature remodeling [7–10] and insulin sensitivity [11], and is also up-regulated in a variety of cancers [12–14]. While relatively little is known about the specific molecular mechanisms by which DCBLD2 transduces or modulates signals, DCBLD2 has been shown to both positively and negatively regulate receptor tyrosine kinase (RTK) signaling [7, 9, 11]. DCBLD2-deficient mice show lower blood glucose levels and increased insulin-induced activation of MAPK and Akt [11]. In cultured vascular smooth muscle cells, DCBLD2 knockdown similarly shows an increase in PDGF-induced stimulation of MAPK [15]. These data suggest that DCBLD2 might normally inhibit RTK signaling. However, VEGF-dependent activation of MAPK in DCBLD2-deficient mice is reduced, suggesting a single mode of action is insufficient to describe how DCBLD2 regulates RTK signaling. One way by which DCBLD2 might regulate insulin and PDGF signaling is by altering the levels of ubiquitin ligases in complex with RTKs as was shown for the insulin receptor (IR). This in turn can regulate the ubiquitination, internalization and degradation of RTKs and therefore their signaling capacity [11, 15]. In VEGF signaling, DCBLD2 was shown to reduce the binding of tyrosine phosphatases to the VEGFR and this may be how DCBLD2 increases VEGF signaling to MAPK [9].

**Figure 1:**
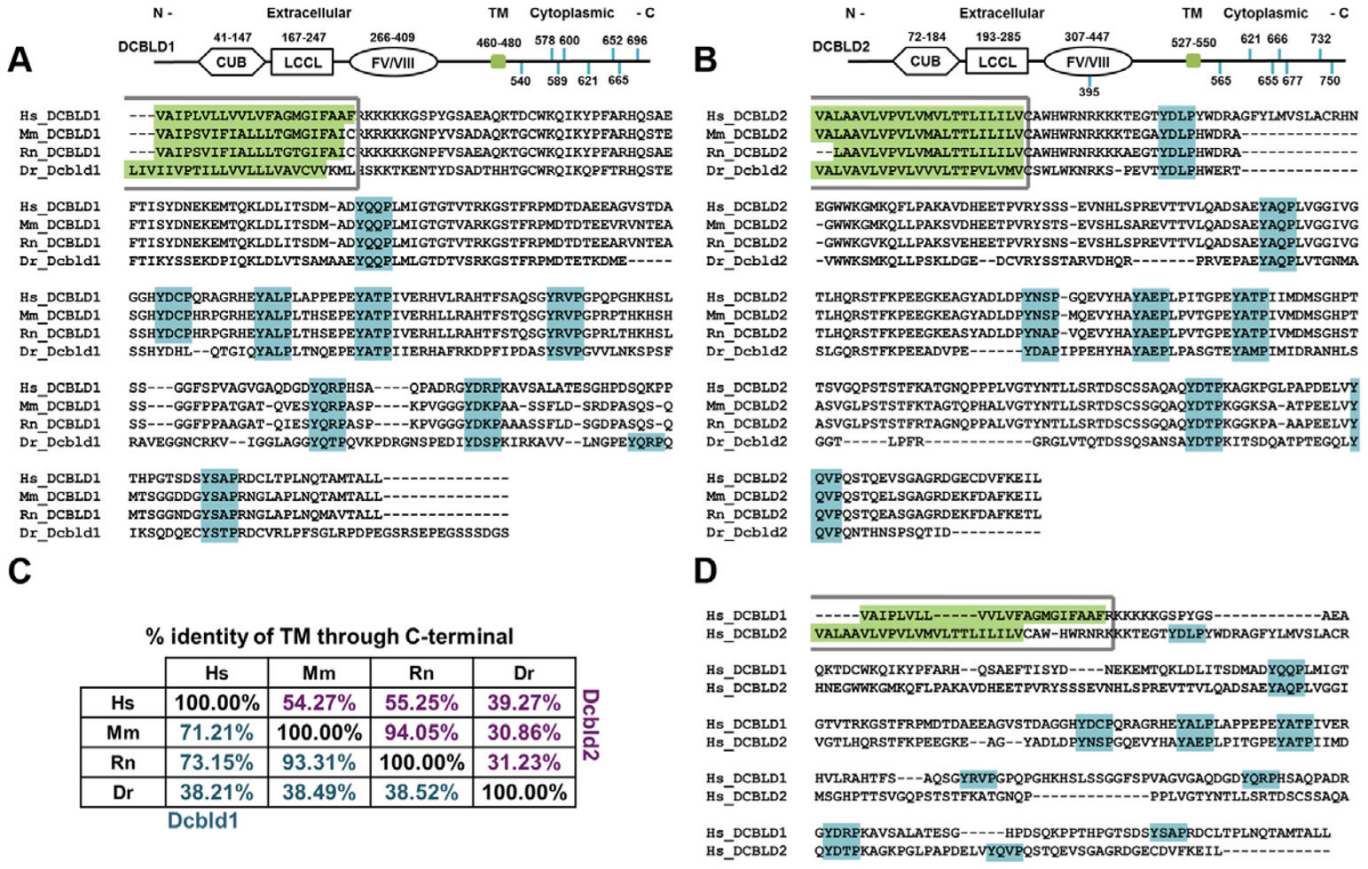
DCBLD1 and DCBLD2 YxxP sites are conserved across vertebrates and family members. Domain structure, YxxP motif locations (blue), and alignments of transmembrane (green) through C-terminal sequences of (A) DCBLD1 and (B) DCBLD2 from four model vertebrates (Hs = human, Mm = mouse, Rn = rat, Dr = zebrafish) are shown below with percent identity of amino acids tabulated in (C). Further, five YxxP sites are conserved between human DCBLD1 and DCBLD2 (D).

In the mechanisms described above, the roles of DCBLD2 tyrosine phosphorylation were not explored. However, in the case of oncogenic EGFR signaling, DCBLD2 was shown to be phosphorylated at Tyr750 thereby activating a TRAF6-Akt pathway [7]. TRAF6 binding to DCBLD2 requires phosphorylation of Tyr750 in a PxExxY motif [7]. This motif at Tyr750 is conserved in DCBLD2 across many, but not all vertebrates. On the other hand, a more striking motif conservation is embodied by DCBLD2’s seven intracellular YxxP motifs. These YxxP motifs are not only highly conserved among DCBLD2 orthologs, but they are also highly conserved in their related paralog, DCBLD1, which harbors eight YxxP motifs (Figure 1). While to date almost all studies involving DCBLD2 have importantly focused on the interaction of DCBLD2 with RTKs, we have proposed that the clustering of DCBLD proteins by unknown ligands could be an important RTK-independent mechanism of DCBLD signaling [6]. In order to understand how these conserved motifs might participate in non-RTK or RTK signaling pathways it is important to establish the contribution of specific kinases to the phosphorylation of specific YxxP motifs on DCBLD proteins. In this study we used biochemical methods and several quantitative mass spectrometry approaches to characterize the phosphorylation of DCBLD proteins by the non-receptor tyrosine kinases of the Src and Abl families, both known to specifically target YxxP motifs [16], and both known to be activated by both RTK-dependent and RTK-independent signaling mechanisms [17–21]. We also evaluated how well phosphorylation by SFKs and Abl family kinases induces the interaction of DCBLD proteins with the SH2 domain of CRKL. Our results uncovered distinct site-specific differences in the regulation of DCBLD1 and 2 by SFKs and Abl kinases.

## EXPERIMENTAL

### Multiple sequence alignments

Multiple sequence alignments were constructed with the UniProt Align tool (EMBL-EBI, Cambridge, United Kingdom; SIB Swiss Institute of Bioinformatics, Geneva, Switzerland; Protein Information Resource, Washington, DC, USA). Conservation of intracellular amino acids and YxxP motifs was examined across representative model vertebrates: DCBLD1 *homo sapiens* (Hs, Q8N8Z6), *mus musculus* (Mm, Q9D4J3), *rattus norvegicus* (Rn, D3ZFM7), *danio rerio* (Dr F1QGI1); DCBLD2 Hs (Q96PD2), Mm (Q91ZV3), Rn (Q91ZV2), Dr (B0S5V9).

### Materials

Penicillin/Streptomycin 100X solution and Dulbecco’s Modified Eagle Medium (DMEM) were obtained from Mediatech (Manassas, VA, USA). SILAC media and kanamycin sulfate were acquired from Thermo Scientific (Waltham MA, USA), and fetal bovine serum (FBS), dialyzed FBS for SILAC experiments, and cosmic calf serum (CCS) were purchased from Hyclone (Logan, Utah, USA). Heavy labeled L-arginine (^13^C_6_, ^15^N_4_) and L-lysine (^13^C_6_, ^15^N_2_) were obtained from Cambridge Isotope Laboratories Inc. (Tewksbury, MA, USA), and unlabeled L-arginine (^12^C_6_, ^14^N_4_) and L-lysine (^12^C_6_, ^14^N_2_) were purchased from MP Biomedicals (Santa Ana, CA, USA). The ProFection® Mammalian Transfection System kit for calcium phosphate transfections and the trypsin used in enzymatic digests prior to LC-MS/MS analysis were from Promega (Madison, WI, USA). Calf Intestinal Phosphatase (CIP) was obtained from New England Biolabs (Ipswich, MA, USA). The SFK inhibitor Src-1 was purchased from EMD-Calbiochem (Billerica, MA, USA) and Imatinib (STI571), the Abl-specific inhibitor, was acquired from Selleckchem (Houston, TX, USA). The BSA standard for Bradford assays and the Bradford Reagent were obtained from Amresco Life Sciences, LLC. (Cleveland, OH, USA). For the development of Western blots, enhanced chemiluminescence (ECL) reagents were purchased from Pierce (Rockford, IL, USA), and x-ray film was from Denville scientific (Metuchen, NJ, USA). Synthetic stable isotope-labeled peptide standards (SL peptides) were from Cell Signaling Technologies (Danvers, MA, USA). Packing material used for HPLC was 5 μm C18-coated silica beads, 200 Å pore size, purchased from Michrom Bioresources Inc. (Auburn, CA, USA). Nitrocellulose membranes were from GVS Life Sciences (Sanford, ME, USA). All additional reagents were purchased from Sigma (St. Louis, MO, USA) unless otherwise noted.

### Plasmids

Mammalian expression constructs for full-length DCBLD2 (Hs) and DCBLD1 (Mm) in pCMV6-Entry, tagged with Flag and Myc sequences at C-termini, were obtained from Origene (#RC224483 and #MR206887 respectively; Rockville, MD, USA). Wild-type (WT) FYN [22] and kinase dead (K299M) FYN [22] plasmids in pRK5-Entry were acquired from AddGene (Cambridge, MA, USA). The human c-Abl construct, with a C-terminal Flag tag was kindly gifted by A. Howe (U. of Vermont), originally constructed in the Kufe lab (Harvard Medical School) [23]. DCBLD2 mutant constructs: DCBLD2-Y1F (Tyr750Phe), DCBLD2-Y3F (Tyr750Phe, Tyr732Phe, Tyr565Phe), and DCBLD2-Y7F (Tyr750Phe, Tyr732Phe, Tyr677Phe, Tyr666Phe, Tyr655Phe, Tyr621Phe, Tyr565Phe) were described previously [6]. The C-terminal Myc- and Flag-tagged DCBLD1 WT and mutant DCBLD1-Y8F (Tyr540Phe, Tyr578Phe, Tyr589Phe, Tyr600Phe, Tyr621Phe, Tyr652Phe, Tyr665Phe, and Tyr696Phe) constructs in pCMV6 vectors were synthesized by Bio Basic Inc. (Markham, ON). The bacterial expression plasmids encoding the fusion of glutathione S-transferase with the CRKL-SH2 domain (GST-CRKL-SH2) and GST-ABL-SH2 were kindly gifted by A. Imamoto (U. of Chicago) and S. Kornbluth (Duke U.), respectively.

### Antibodies

The mouse α-Flag (M2) antibody (Ab) and Affinity Gel were from Sigma and the free Ab was used for Western blotting at a concentration of 0.5 μg/mL. Cell Signaling Technologies Inc. (Danvers, MA, USA) was the source of the following antibodies, used at the indicated dilution or concentration: α-Flag (M2, rabbit mAb; 1:1000), α-Fyn (rabbit mAb; 1:2000), α-Src (rabbit mAb; 1:2000), α-pTyr416-Src (rabbit mAb; 1:5000), α-pTyr412-Abl (rabbit mAb; 1:1000), α-alpha-tubulin (1:1000), and α-pan-actin (0.1 μg/mL). The rabbit α-Abl antibody (K-12, 0.2 μg/mL) was from Santa Cruz Biotechnology (Dallas, TX, USA). The α-phosphotyrosine (4G10; 1:1000) was from EMD Millipore (Billerica, MA, USA) and the α-Myc (9E10; 1:1000) was obtained from American Type Tissue Collection (Manassas, VA, USA). All primary antibodies were diluted in 1.5% BSA in Tris-Buffered Saline (0.9% NaCl, 0.4% Tris-HCl, and 0.1% Tris-base) with 0.05% Tween 20 (TBS-T) and containing 0.005% sodium azide. Horseradish peroxidase (HRP)-conjugated secondary antibodies were obtained from EMD Millipore and used at the following concentrations: goat α-mouse IgG-HRP (1:5,000), light-chain-specific goat α-mouse IgG-HRP (1:10,000) and goat α-rabbit IgG-HRP (1:15,000). All secondary antibodies were diluted in TBS-T.

### Cell culture, transfection, inhibitors, stimulation, and cell lysis

Adenovirus early region-transformed HEK 293 cells were cultured in DMEM supplemented with 5% each of FBS and CCS, 50 U/mL penicillin, and 50 μg/mL streptomycin at 37 °C in 5% atmospheric CO_2_. For experiments involving stable isotope labeling of amino acids in cell culture (SILAC), HEK 293 cells were cultured in labeled (heavy) or unlabeled (light) growth medium for at least one week prior to transfection to ensure full incorporation of stable isotopes into proteins. SILAC media, lacking L-lysine and L-arginine were supplemented with 10% dialyzed FBS and antibiotics as stated above, 60 mg/L unlabeled L-proline, 100 mg/L of L-lysine either unlabeled or labeled (^13^C_6_, ^15^N_2_), and 100 mg/L of L-arginine either unlabeled or labeled (^13^C_6_, ^15^N_4_).

HEK 293 cells were grown to 60% of confluence prior to transfection via calcium phosphate precipitation. The following amount of plasmid was transfected per 10 cm dish: wild type and mutant DCBLD1 and DCBLD2 (6 μg), wild type FYN (1.5 μg), kinase-dead FYN (2.5 μg), and wild type c-ABL (2 μg). Six hours post-transfection, cells were washed with phosphate-buffered saline (PBS) and returned to full medium overnight before lysis. Cells treated with inhibitors received 2 μM Src-1 and/or 20 μM STI571 in dimethyl sulfoxide (DMSO) for 15 min at standard culture conditions, prior to stimulation with 8.8 mM H_2_O_2_ (15 min, with or without inhibitor treatment). Cells were placed on ice immediately following H_2_O_2_ stimulation and washed with PBS (4 °C) prior to lysis in Brain Complex Lysis Buffer (BCLB: 25 mM Tris pH 7.2, 137 mM NaCl, 10% glycerol, 1% igepal, 25 mM NaF, 10 mM Na_2_H_2_P_2_O_7_, 1 mM Na_3_VO_4_, 1 mM phenylmethylsulfonyl fluoride (PMSF), 10 μg/mL each leupeptin and pepstatin-A). Lysates were centrifuged and the supernatant was reserved and stored at −20 °C for further analysis.

### Immunoprecipitation, Western blotting and SDS-PAGE

Protein concentration was determined using an Eppendorf BioPhotometer Plus (Eppendorf; Hamburg, Germany) with BSA standards. For immunoprecipitations, normalized lysates (10^3^ μg total protein in 750 μL) were incubated with α-Flag Affinity Gel (10 μL of a 50 % slurry in BCLB) overnight, rocking at 4 °C. The beads were washed four times with BCLB, after which bound proteins were eluted and denatured in 25 μL sample buffer (150 mM Tris pH 6.8, 2% SDS, 5% β-mercaptoethanol, 7.8% glycerol, 0.25 ng/mL bromophenol blue) at 95 °C for 5 min. For Western blotting of whole cell lysates, samples were denatured in sample buffer and 15 μg total protein was loaded per lane. Immunoprecipitations, pull-downs, whole cell lysates, and phosphatase assays were separated on 10% acrylamide gels (30% w/v and 37.5:1 acrylamide:bis-acrylamide) with 4.2% acrylamide stacking gels. Current was maintained at 20 mA and 30 mA per gel through the stacking and separating layers, respectively. Following separation, proteins were either transferred to a nitrocellulose membrane for Western blotting or stained with Coomassie for mass spectrometric analysis. Transfers to nitrocellulose membranes were in a submersible transfer unit at 4 °C (400 mA for 2 hours) in 1.13% glycine, 0.25% Tris-base and 20% methanol. Membranes were stained with a reversible Ponceau stain (0.5% Ponceau and 1% acetic acid in H_2_O) to assess total protein levels and were then blocked with 5% non-fat dry milk in TBS-T. Primary antibody solutions were incubated overnight at 4 °C. Membranes were then washed three times with TBS-T and incubated with HRP-conjugated secondary antibody solution for three hours at 25 °C. After three final washes in TBS-T, membranes were briefly incubated in ECL reagents and exposed to x-ray film

### Phosphatase assay

Anti-Flag resin was added to cell extracts containing 1 mg of protein in 500 μL BCLB as described above. After washing three times with BCLB and once with PBS, the resin from each immunoprecipitation was divided equally into two separate tubes and drained. Resins were resuspended in 50 μL Calf Intestinal Phosphatase (CIP) Buffer (100 mM NaCl, 1 mM Tris-HCl pH 8.0, 10 mM MgCl_2_, 1 mM 1,4-dithiothreitol) with or without 2.5 units of CIP, after which all samples were incubated at 37 °C on a dry block for 3 hours. Proteins were eluted and denatured at 95 °C after the addition of 4x protein sample buffer. Denatured proteins were prior subjected to SDS-PAGE and Western blotting.

### GST-CRKL-SH2 pull-down assay

To generate GST-CRKL-SH2-conjugated glutathione beads, 50 mL cultures of Luna Broth (LB; 0.5% Tryptone, 0.5% yeast extract, 171 mM NaCl) containing 50 μg/mL ampicillin were inoculated with *E. Coli* harboring a plasmid encoding GST-CRKL-SH2 and incubated overnight at 37 °C, shaking at 250 rpm. This initial culture was then spiked into 500 mL LB with ampicillin, and incubation was continued for 2 hours, at which time expression was induced by the addition of isopropyl β-D-1-thiogalactopyranoside (IPTG) to 1 mM, followed by an additional 4 hours of incubation. Cultures were then centrifuged at 4 °C (6,000 × g, 20 min). The supernatants were decanted and the pellets were stored at −20 °C. Pellets were resuspended in 10 mL Bacterial Lysis Buffer (BLB; 100 mM ethylenediaminetetraacetic acid, 1 mM PMSF, 10 μg/mL each leupeptin and pepstatin-A in PBS). Cells were sonicated in six 30 sec intervals, intermitted with equal rest periods on ice, after which 1 mL of 10% Triton X-100 was added. Lysates were mixed and centrifuged at 4 °C (12,000 × g, 20 min) and pellets were discarded. Glutathione resin (300 μL of a 50% slurry in BLB) was added to the GST-CRKL-SH2-enriched supernatant and rocked overnight at 4 °C. Beads were washed three times in BLB, once in BCLB, and twice in PBS and then stored in PBS at 4 °C. For pull-down assays, HEK 293 cell lysates were rocked with 15 μL GST-CRKL-SH2-conjugated glutathione beads (30% slurry in PBS) at 4 °C. Beads were washed three times with BCLB and proteins were eluted and denatured in 25 μL of sample buffer at 95 °C for 5 min prior to analysis via SDS-PAGE and Western blotting. GST-ABL-SH2-conjugated beads were obtained using the protocol outlined above, after which the purified protein was eluted with 10 mM glutathione.

### Peptide preparation, mass spectrometry, data analysis and statistics

Bands containing immunoprecipitated proteins of interest were excised from Coomassie-stained acrylamide gels, diced to 1 mm cubes and transferred to separate microcentrifuge tubes. Gel pieces were washed with HPLC-grade H_2_O and then de-stained in 50 mM ammonium bicarbonate (NH_4_HCO_3_) and 50% acetonitrile (MeCN) at 37 °C for 30 minutes. De-stain was then removed and gel pieces were dehydrated in 100% MeCN. On ice, dried gel pieces were rehydrated in 25 μL of 12 ng/μL sequencing grade modified trypsin in 50 mM NH_4_HCO_3_. After a 20-minute incubation at 4 °C, an additional 25–50 μL of 50 mM NH_4_HCO_3_ were added to submerge gel pieces, followed by overnight incubation at 37 °C. After centrifugation, supernatants were transferred to new microcentrifuge tubes. Remaining peptides were extracted from gel pieces with the addition of 50% MeCN, 2.5% formic acid (FA). Supernatants were combined with the initial tryptic digest supernatants, and gel pieces were dehydrated in 100% MeCN. The final extraction was combined with the previous two extractions and peptides were dried in a speed-vac.

Peptides were resuspended in 2.5% MeCN/0.15% FA (Solvent A) and separated on a reverse-phase HPLC column (length=12 cm × 100 μm) packed in house with 5 μm C18 beads (pore size=200 Å) prior to analysis via a linear ion trap-orbitrap (LTQ-Orbitrap Discovery; resolution=3×10^4^, scan speed=1 Hz) mass spectrometer fitted with a Finnigan Surveyor Pump Plus and Micro AS autosampler (Thermo Electron; San Jose, CA, USA) and controlled with Xcalibur^™^ 2.1 Software (Thermo Fisher Scientific, Inc.; Waltham, MA, USA). Following a 15 min loading phase (flow rate=100 μL/min) onto the C18 column in Solvent A, peptides were eluted using a 0–50% gradient of Solvent B (99.85% MeCN, 0.15% FA) over 38 min and electrosprayed (2.1 kV) into the mass spectrometer. This gradient was followed by 7 min at 100% Solvent B before a 10 min equilibration in 100% Solvent A.

The precursor scan (360–1700 *m/z*) was followed by ten low energy collision-induced dissociation (CID) tandem mass spectra. CID spectra were acquired for the top two most abundant ions in the precursor scan (dynamic exclusion settings: repeat count=2, repeat duration=30 sec, exclusion list size=100, exclusion duration=60 sec, exclusion width= ±1.5 *m/z*) followed by eight targeted scans for DCBLD1 or DCBLD2 peptides of interest (Supplementary Tables 1 and 2). Target mass isolation windows were set to ±1.6 *m/z* for *z* of +2, and 0.9 *m/z* for *z* of +3. All mass spectra were obtained in centroid with precursor ion spectra acquired in the orbitrap and fragment ion spectra in the linear ion trap.

SEQUEST searches were performed using a forward and reverse 2011 Uniprot Human Protein database requiring tryptic peptides and permitting the following modifications: phosphorylation of serine, threonine and tyrosine (+79.9663 Da), oxidation of methionine (+15.9949 Da), and acrylamidation of cysteine (+71.0371 Da). Mass additions in experiments employing isotopically labeled residues were as follows: heavy lysine (+8.0142 Da) and arginine (+10.0083 Da) in SILAC analyses, and heavy leucine (+7.0172 Da) and valine (+6.0138 Da) when using SL peptides.

Peptides were manually quantified in Xcalibur using the precursor scan monoisotopic peak intensities averaged across full width at half maximum (FWHM) of the elution window. In the case of SILAC and SL peptides, the heavy-to-light ratio (H:L) or L:H of monoisotopic peak intensities, for SILAC or SL quantification respectively, were determined. Ratios were normalized to reference peptides of DCBLD1 or DCBLD2 that were not found modified (Supplementary Table 3A,B). For SILAC experiments, kinases were co-expressed with DCBLD proteins in cells grown in medium supplemented with stable-isotope labeled argmine and lysine and paired with a light culture expressing the DCBLD proteins alone. In the case of WT FYN / DCBLD(X) co-expression, the corresponding light culture possessed kinase-dead (KD) FYN co-transfected with DCBLD(X). Inhibitors were applied to the light cultures prior to H_2_O_2_-stimulation and paired with heavy cultures subjected to H_2_O_2_-stimulation alone. Heavy and light samples were immunoprecipitated separately and eluted proteins were combined as heavy and light pairs before SDS-PAGE.

Each set of SL peptides or label-free (LF) experiments had six conditions with three replicates for each condition as follows: **a)** mock, **b)** DCBLD(X), **c)** DCBLD(X) with H_2_O_2_, **d)** DCBLD(X) with WT FYN or Src-1/H_2_O_2_, **e)** DCBLD(X) with c-ABL or STI571/H_2_O_2_, **f)** DCBLD(X) with WT FYN/c-ABL or Src-1/STI571/H_2_O_2_. Approximately 300 fmol of each SL peptide (Supplementary Table 2A) were spiked into tryptic peptide samples. These included peptides corresponding to the tryptic peptides harboring unphosphorylated and phosphorylated DCBLD2 peptides, as well as a DCBLD2 peptide (GFLASYSVIDK) that was not found post-translationally modified as a reference for relative DCBLD2 levels. The L:H value for this reference peptide was used to normalize all L:H ratios from SL peptides (Supplementary Table 3B). In label-free (LF) quantification, approximate local noise levels were subtracted from monoisotopic intensities and then divided by the average ion intensities of reference peptides (Supplementary Table 3A,B). Reference intensities in this method were obtained from an average of three unmodified peptides (LNSNEVTVLFK, GSHYFEEK, and DIAGDISGNTK for DCBLD1; IYNGIGVSR, NNFLPPIIAR, and FTQPLQPR for DCBLD2; *m/z* tabulated in Supplementary Table 3A,B) regularly identified in each LC-MS/MS run.

To quantify changes in phosphorylation in the presence of H_2_O_2_, kinases, or inhibitors, normalized L:H (SL peptides) or LF intensities were divided by the condition in which we predicted to find to the maximum signal per peptide. It was predicted that the maximum number of unphosphorylated peptides would be found in the unstimulated condition, and that of phosphorylated peptides would be observed in the co-expression of Fyn and c-Abl with DCBLD(X) in kinase expression experiments, or in the H_2_O_2_-stimulated condition in the case of inhibitor treatments. Changes in the number of observed ions across conditions were reported as a percent relative to the condition that would yield the predicted maximum. The percent change relative to the treatment deemed the predicted max was determined as described above. Two and three trials were conducted for each condition with DCBLD1 and DCBLD2, respectively.

Quantified changes in phosphorylation state among experimental conditions were analyzed for significance with a one-way ANOVA test. The Tukey-HSD (*P* < 0.05) was used to compare each experimental condition to the others. Methods of SL peptides and LF quantification for a given peptide and experimental condition were compared using a t-test (*P* < 0.05), with the exception of Tyr565. Four distinct methods of quantification were considered for the peptide harboring Tyr565 due to alternative cleavage patterns and the use of both SL and LF methods, necessitating the use of a Tukey-HSD (*P* < 0.05) analysis. The four methods used to quantify phosphorylation at this site were compared using a one-way ANOVA and Tukey-HSD (*P* < 0.05). Statistical analyses were performed in JMP Pro 12 Statistical Software (SAS Institute, Cary, NY, USA).

## RESULTS

### DCBLD1 and DCBLD2 YxxP motifs are highly conserved across vertebrates

Previously, we identified tyrosine phosphorylation sites in YxxP motifs within the DCBLD2 intracellular domain and found that these motifs were essential for the phosphorylation-dependent binding of the signaling adaptor CRKL [6]. Figure 1 displays the extracellular domain structure, including CUB, LCCL, and FV/FVIII domains, as well as intracellular tyrosines in YxxP motifs within human DCBLD1 and DCBLD2. Also shown are multiple sequence alignments of the TM domain through the C-terminus for each family member across model vertebrates. Percent identity of the intracellular sequences of each protein for the aligned species is shown in Figure 1C. Both paralogs have eight YxxP motifs, all of which are intracellular with the exception of one from DCBLD2. Importantly, while the overall conservation of the intracellular domains of DCBLD1 and 2 between mammals and zebrafish deviate considerably (~40% identity), the same number of intracellular YxxP motifs can be found across the aligned sequences with only one YxxP position in zebrafish falling out of alignment (Figure 1A). A similar analysis of the TM through C-terminal domains of human DCBLD1 and 2 was conducted to examine sequence and motif conservation within the protein family. While there appears to be little homology between other intracellular amino acid sequences, five YxxP sites between DCBLD1 and 2 are conserved (Figure 1D). This striking conservation of YxxP motifs suggests that this feature is essential for the specific signaling roles inherent to this novel family of proteins. Indeed, we previously-reported the importance of DCBLD2 YxxP motifs in the interaction with the CRKL-SH2 domain [6]. This initial discovery led us to investigate if the paralog DCBLD1 might also show a reversible interaction with the CRKL-SH2 domain and to determine the relative contribution of the non-receptor tyrosine kinases of the Abl and SFK family in the phosphorylation of specific DCBLD1 and DCBLD2 YxxP motifs.

### DCBLD1 binds the CRKL-SH2 domain upon induction of tyrosine phosphorylation

**Figure 2:**
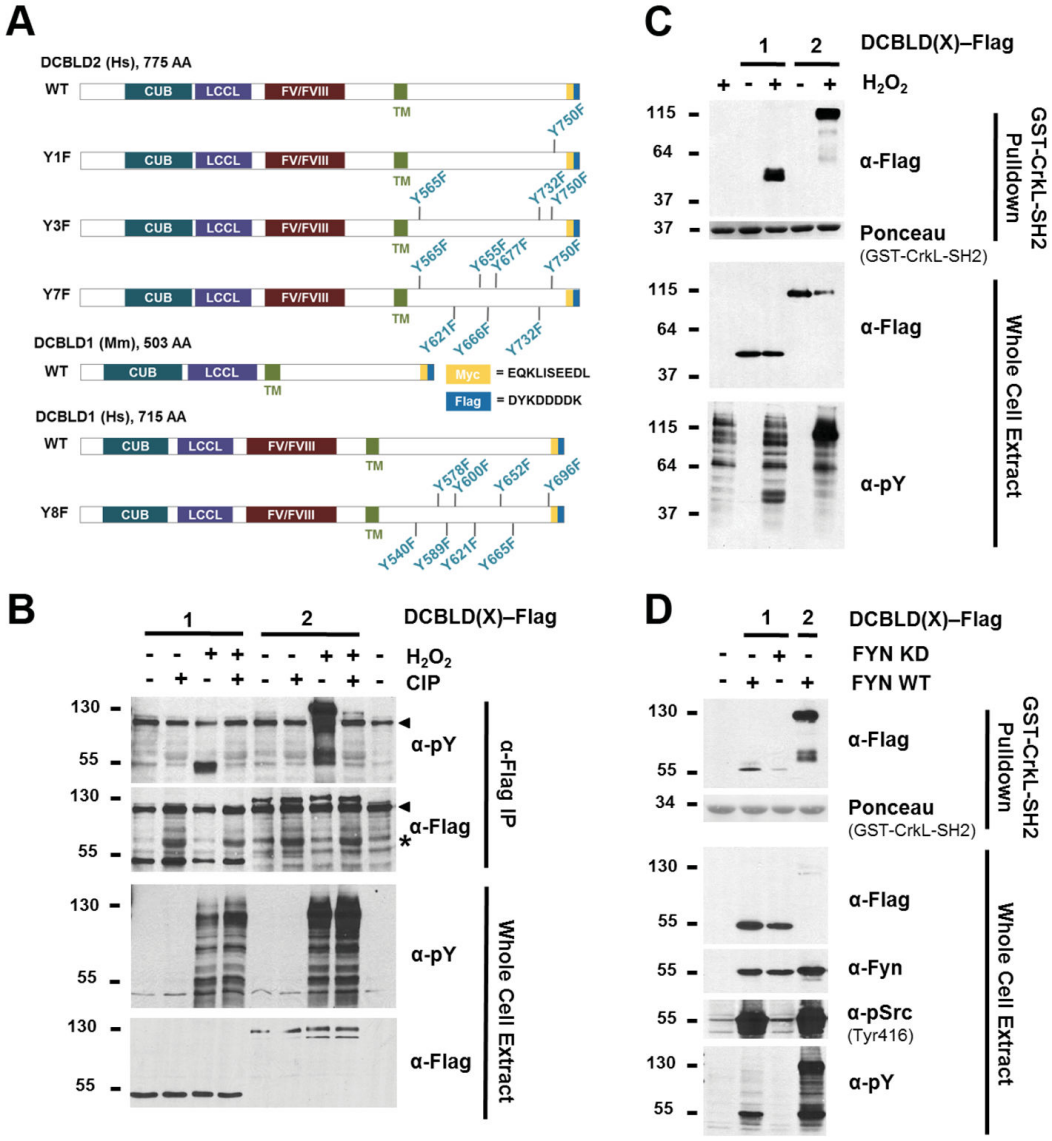
DCBLD1 is reversibly tyrosine phosphorylated and binds the CRKL-SH2 domain following stimulation by H_2_O_2_ and Fyn. (A) Schematic of DCBLD family member expression constructs used in this study. (B) H_2_O_2_ stimulation of HEK 293 cells stimulates endogenous tyrosine kinases to phosphorylate DCBLD1 and DCBLD2. DCBLD1-Flag and DCBLD2-Flag were immunoprecipitated from transfected cells, which had been left untreated or stimulated with H_2_O_2_ for 20 min prior to lysis. Immune complexes were incubated with or without calf intestinal phosphatase (CIP; “*” denotes antibody cross-reaction with CIP) before analysis via SDS-PAGE and Western blotting for anti-phosphotyrosine. Arrows indicate signals from antibody aggregates in the anti-pY and anti-Flag panels of the IP. A light-chain-specific secondary antibody was used to resolve the DCBLD1 signal from that of the heavy chain. (C) H_2_O_2_ stimulation of HEK 293 cells induces DCBLD1 and DCBLD2 to bind to the CRKL-SH2 domain. Pulldown assays (upper panels) using GST-CRKL-SH2 were performed on whole cell extracts (lower panels) from cells expressing Flag-tagged DCBLD(X) and stimulated with or without H_2_O_2_ for 20 min as indicated. The antibodies used for each blot are indicated. The Ponceau stain of the blot prior to anti-Flag immunoblotting is shown to indicate the relative levels of GST-CRKL-SH2 in the pulldowns. (D) Fyn induces DCBLD family members to bind to the CRKL-SH2 domain. Pulldown assays were conducted as described in (B) above. Pulldown assays were stained with Ponceau followed by immunoblotting with anti-Flag. Whole cell extract blots were with the indicated antibodies. The anti-pSrc antibody is cross-reactive will active SFKs including Fyn.

One way to acutely induce tyrosine phosphorylation on cellular substrates by endogenous kinases is to inhibit tyrosine phosphatases by treating cells with H_2_O_2_ [24]. We previously reported that H_2_O_2_ induced tyrosine phosphorylation of DCBLD2 YxxP sites and thereby facilitated the binding of DCBLD2 with the CRKL-SH2 domain [6]. To determine whether DCBLD1 is also tyrosine-phosphorylated in a regulated manner, a phosphatase assay was conducted. Flag-tagged DCBLD1 and DCBLD2 (Figure 2A) were expressed in HEK 293 cells, and cells were H_2_O_2_-stimulated prior to lysis. α-Flag immunoprecipitations (IPs) of the extracts were incubated with or without calf intestinal phosphatase (CIP). Regulated tyrosine phosphorylation on DCBLD1 and DCBLD2 was demonstrated following immunoblotting for phosphotyrosine (Figure 2B), given H_2_O_2_-stimulated tyrosine phosphorylation observed in the WCE was lost when CIP was incubated with the immune complexes. We next tested if H_2_O_2_-induced tyrosine phosphorylation on DCBLD1 would render it capable of binding to the CRKL-SH_2_ domain. A GST-CRKL-SH2 pull-down (PD) was performed with lysates from untreated and H_2_O_2_-stimulated cells expressing either DCBLD1 or DCBLD2. Both paralogs showed H_2_O_2_-induced binding to the CRKL-SH2 domain (Figure 2C).

### YxxP sites are necessary for the interaction of human DCBLD1 with the CRKL-SH2 domain

The initial expression construct we were able to obtain for DCBLD1, and the one we characterize in detail below, was derived from mouse. Interestingly, rodent DCBLD1 sequences are smaller than other vertebrate orthologs as they lack the FV/FVIII portion of the ectodomain. To validate whether human DCBLD1 also showed stimulus-dependent binding to the CRKL-SH2 domain, and to determine if the intracellular YxxP motifs were essential for this interaction, we obtained an expression construct for human DCBLD1 as well as a mutant with all intracellular YxxP motifs mutated to FxxP motifs (DCBLD1 Y8F). WT DCBLD1, but not DCBLD1 Y8F exhibited regulated binding to the CRKL-SH2 domain in response to H_2_O_2_ stimulation (Supplementary Figure 1). These results demonstrate that both human and mouse DCBLD1 orthologs show a similar reversible interaction with the CRKL-SH2 domain and establishes the necessity of the DCBLD1 YxxP sites for the CRKL-SH2 interaction.

### Fyn and Abl variably induce DCBLD1 and DCBLD2 to bind the CRKL-SH2 domain

Our previous characterization of the DCBLD2-CRKL interaction demonstrated that co-expression of the SFK Fyn was sufficient to induce DCBLD2 to bind to the CRKL-SH2 domain [6]. This led us to examine the effect of Fyn activity on the DCBLD1-CRKL interaction. We found that co-expression of DCBLD1 with active Fyn, but not kinase-dead Fyn, was sufficient to induce DCBLD1 binding to the CRKL-SH2 domain in a pull-down assay (Figure 2D). However, the relative amount of DCBLD2 that Fyn induced to bind to the CRKL-SH2 domain was far higher than the amount of DCBLD1 that Fyn induced to bind to the CRKL-SH2 domain (Figure 2D).

The Abl kinase family (Abl and Arg) demonstrate high specificity for tyrosine residues in YxxP motifs [16, 18]. Therefore, the effect of Src-1 [25], a SFK inhibitor, was compared to that of an Abl-specific inhibitor (STI571) [26], on the interaction of DCBLD1 and DCBLD2 with the CRKL-SH2 domain to determine whether the activity of endogenous SFKs and/or Abl is necessary for this interaction. Prior to lysis, cells were treated for 15 min with 2 μM Src-1, 20 μM STI571, or both inhibitors prior to H_2_O_2_ stimulation to investigate whether the presence of SFK or Abl inhibitors counteracted the effect of tyrosine phosphatase inhibition on the pull-down assay. Appropriate inhibitor concentrations were determined from a titration in which endogenous SFK and Abl activities were monitored after treatments of increasing concentrations of Src-1 and STI571, individually (Supplementary Figure 2A). This was paired with a CRKL-SH2 pull-down assay using extracts of DCBLD2-transfected HEK 293 cells in order to determine whether the Abl-specific inhibitor would dampen the binding interaction, as was previously observed with Src-1 [6]. The 2 μM Src-1 treatment sufficiently reduced the fraction of active Src (pTyr416), however, we did not attempt to increase inhibitor dose to eliminate SFK activity due the observed reduction in active Abl (pTyr412) following treatment (Supplementary Figure 2A). Sanguinetti *et al* [27] reported that SFKs (Fyn specifically) play a role in Abl activation in response to reactive oxygen species, which may account for the decrease in Abl activity upon SFK inhibition. Although the DCBLD2-CRKL interaction was somewhat affected by the 2 μM Src-1 and 5, 10, and 20 μM STI571, each inhibitor alone was not sufficient to eliminate the binding of DCBLD2 to the CRKL-SH2 domain, suggesting that more than one kinase is capable of mediating the DCBLD2-CRKL interaction. We hypothesized that simultaneous inhibition of SFKs and Abl may be sufficient to eliminate or significantly reduce this interaction. Additionally, we observed an increase in endogenous Abl activity and, to a lesser extent, SFK activity upon H_2_O_2_ stimulation alone in DCBLD2-transfected cells over control cells (Supplementary Figure 2A), which was eliminated with the DCBLD2-Y7F mutant (Supplementary Figure 2B). We hypothesized that this increase in Abl activity above that in untransfected cells stimulated with H_2_O_2_ was the result of a phosphorylation-dependent binding interaction between the Abl-SH2 domain and at least one of the DCBLD2 YxxP motifs. Therefore, we compared the binding capacity of a GST-ABL-SH2 fusion protein with immunoprecipitated DCBLD2-WT and DCBLD2-Y7F (Supplementary Figure 2C). Significantly higher levels of the GST-ABL-SH2 fusion protein bound DCBLD2-WT following H_2_O_2_ treatment, as observed in the anti-GST panel of the IP, suggesting that phosphorylated YxxP motifs are required for the DCBLD2-Abl-SH2 interaction to occur. These data suggest that phosphorylation of DCBLD2 YxxP sites elevate Abl activity through a binding interaction between pYxxP motifs and the Abl-SH2 domain, maintaining the kinase in a catalytically active conformation.

**Figure 3:**
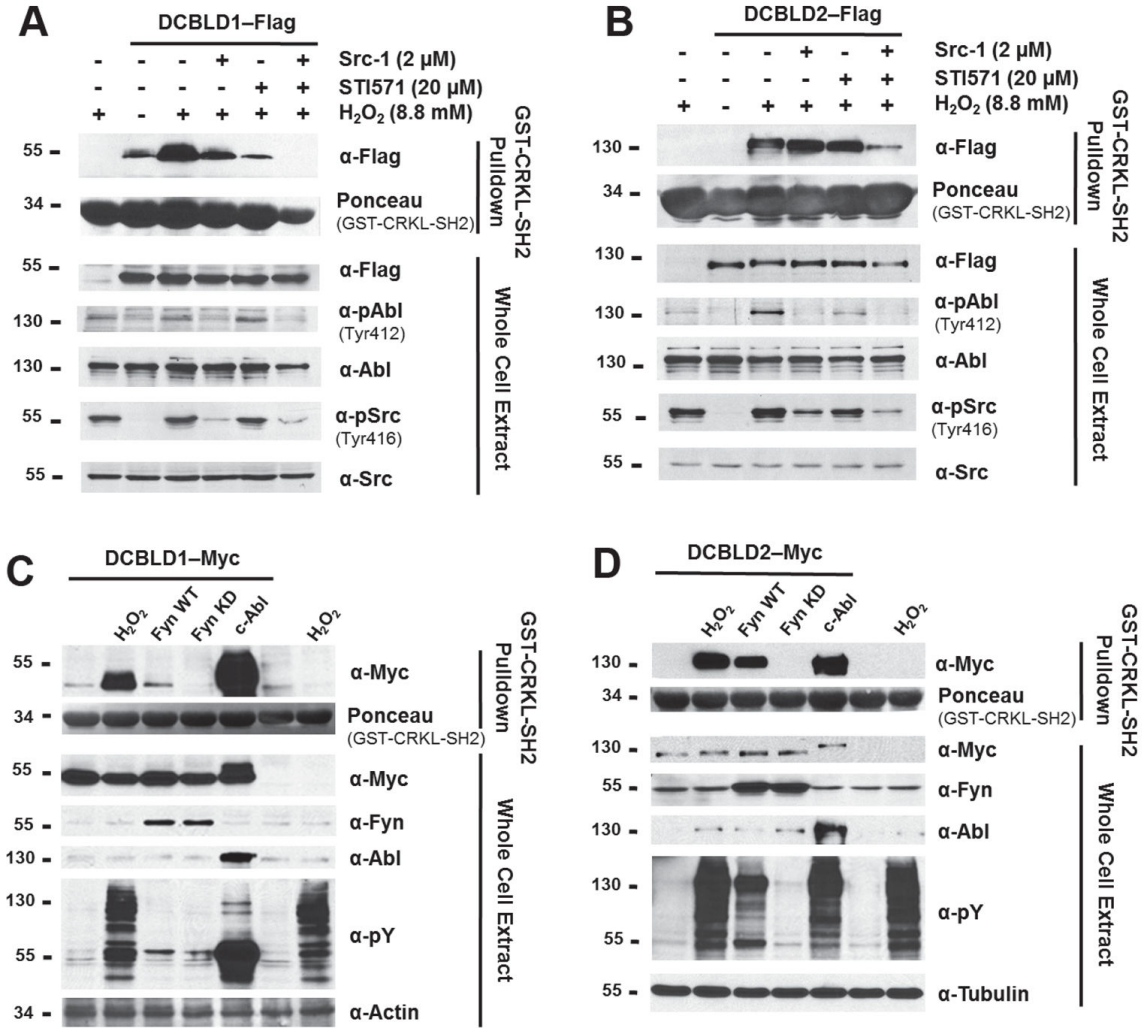
SFKs and Abl are necessary and sufficient to induce DCBLD1 and DCBLD2 to bind to the CRKL-SH2 domain. Pulldown assays were performed from extracts of DCBLD1- and DCBLD2-transfected cells as described in the legend to Figure 2 above. (A) DCBLD1 binding was reduced upon treatment with the SFK inhibitor (Src-1). However, the Abl-specific inhibitor (STI571) more strongly disrupted the interaction. The application of both inhibitors abolished the interaction. (B) No significant reduction in the binding between DCBLD2 and the CRKL-SH2 domain was observed in response to either inhibitor alone. However, the application of both inhibitors strongly reduced the DCBLD2-CRKL-SH2 interaction. To determine whether these kinases were sufficient to induce this interaction, Fyn and c-Abl were co-transfected with (C) DCBLD1 or (D) DCBLD2. While Fyn was able to induce a small degree of binding between the CRKL-SH2 domain and DCBLD1, Abl was superior in this regard.

We then tested the combined effect of Src-1 (2 μM) and STI571 (20 μM) treatment on the binding of CRKL-SH2 to DCBLD1 and DCBLD2 (Figure 3A,B). Src-1 significantly reduced the DCBLD1-CRKL-SH2 interaction in comparison to H_2_O_2_ stimulation alone, but did not reduce binding to the baseline levels observed in the unstimulated condition. However, STI571 was sufficient to reduce binding to baseline levels. Combined treatment of Src-1 and STI571 eliminated the interaction, suggesting that SFK and Abl activities both promote the DCBLD1-CRKL-SH2 interaction. Similarly, while inhibition of SFKs and Abl alone were not sufficient to disrupt the DCBLD2-CRKL-SH2 interaction (Figure 3B), the combination of Src-1 and STI571 significantly reduced the binding. The residual binding upon application of both inhibitors may be due to the incomplete inhibition of SFKs in this experiment, observable in the α-pSrc (Tyr416) blot (Figure 3B).

Upon establishing that activities of endogenous SFKs and Abl are necessary for the H_2_O_2_-induced interaction between the CRKL-SH2 domain and DCBLD proteins, Abl co-expression with DCBLD1 and DCBLD2 was investigated to determine sufficiency while using Fyn as a positive control as established in Figure 2D. Figures 3C and 3D display the CRKL-SH2 pull-downs from extracts of cells co-expressing c-Abl and DCBLD1 or DCBLD2. Here, the Myc epitope tags were employed for immunoblotting for DCBLD proteins, as the c-Abl construct used also possesses a Flag tag and c-Abl-Flag runs at the approximate MW of DCBLD2-Myc-Flag. The pull-down assay from lysates containing DCBLD1 revealed intense phosphorylation by Abl in the whole cell extract (WCE), consistent with the strong signal from the α-Myc blot in the CRKL-SH2 pull-down. Although the specific activities of Fyn and Abl are not identical in this system, it appeared that the effect of Fyn co-expression was reduced relative to H_2_O_2_ stimulation or Abl in inducing DCBLD1 and DCBLD2 to bind to CRKL-SH2. Interestingly, co-expression of DCBLD proteins with Abl significantly reduced the mobility of both DCBLD family members in SDS-PAGE. This could be the result of dramatically higher levels of phosphorylation on DCBLD proteins generally, Abl-specific phosphorylation events, or additional Abl-specific modifications. Fyn and Abl both can drive DCBLD family members to bind to the CRKL-SH2 domain. However, these kinases demonstrate preferences with the binding of DCBLD1 more strongly affected by Abl, suggesting that SFKs and Abl differentially target DCBLD protein YxxP sites.

### SFKs and Abl differentially phosphorylate DCBLD1 and DCBLD2 at distinct tyrosine phosphorylation sites

In order to identify sites that may be differentially regulated by SFKs and Abl, we immunoprecipitated DCBLD1 or DCBLD2 from HEK 293 cell lysates under kinase active or inhibited conditions and monitored the phosphorylation of several of the eight DCBLD1 and seven DCBLD2 intracellular YxxP sites (Supplementary Tables 1–3) using targeted liquid chromatography (LC) tandem mass spectrometry (MS/MS) (Supplementary Figure 3). We also monitored the DCBLD2 peptide harboring Tyr715, a non-YxxP tyrosine residue, as this site was found phosphorylated in a previous analysis [6]. Taking a three-pronged approach for quantification, we were able to monitor changes in phosphorylation at three YxxP sites for each DCBLD protein while comparing the effectiveness of quantitative LC-MS/MS methodologies. Full amino acid sequences and coverage maps for DCBLD1 and DCBLD2 can be found in Supplementary Figures 4A and 4B.

**Figure 4:**
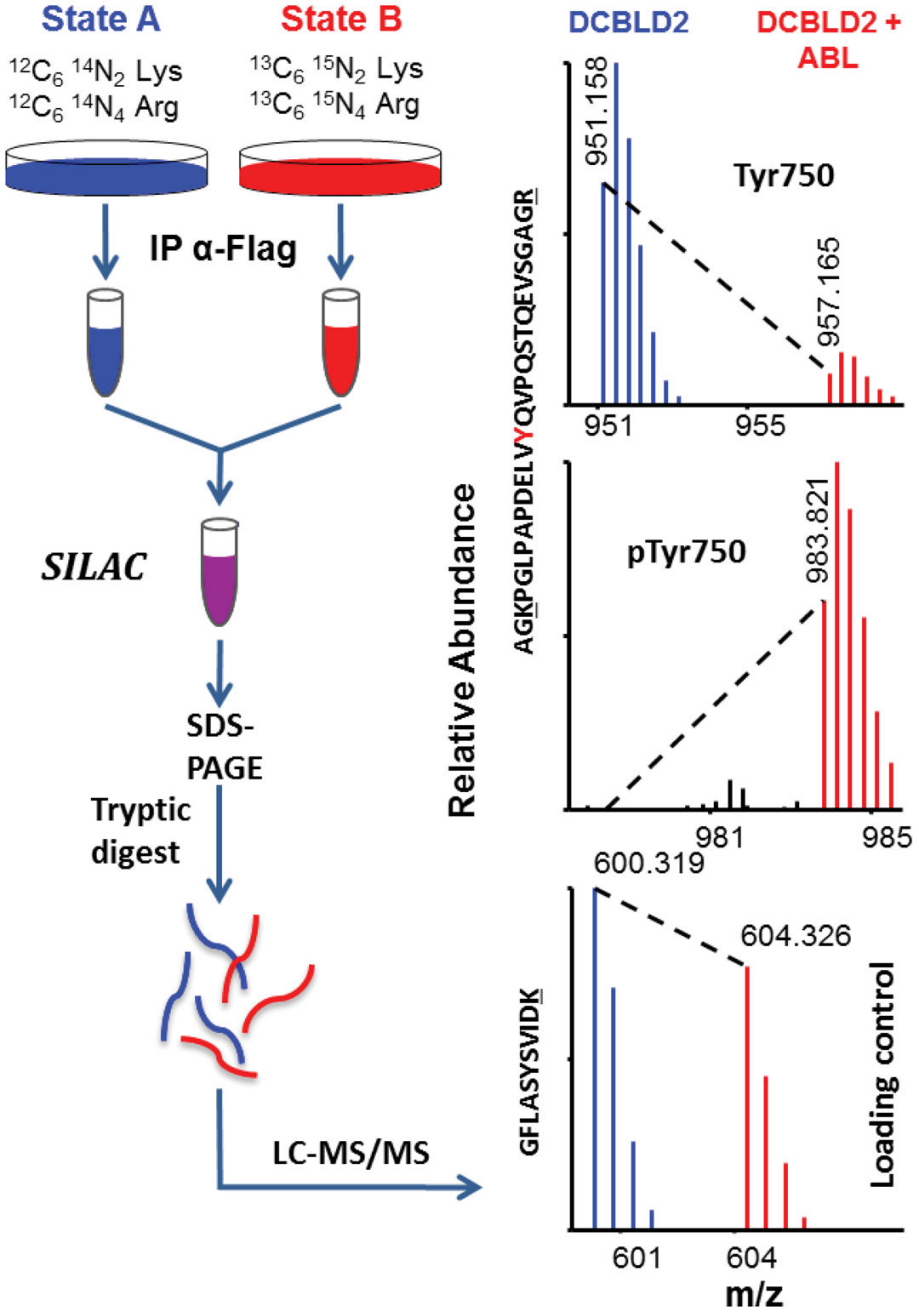
Schematic of sample preparation for SILAC quantification and representative example of quantification by LC-MS/MS. Cells grown in media supplemented with either light or heavy lysine and arginine were transfected with DCBLD(X)–Flag in kinase inactive/inhibited (light) or active (heavy) conditions. Post-IP, heavy and light immune complex pairs were combined and analyzed via LC-MS/MS to determine relative abundance of phosphopeptide ions between each state. Heavy-to-light ratios (H:L) of monoisotopic peak intensities were normalized to DCBLD(X) peptides that were found not to be modified (loading control).

SILAC was used as an initial survey of differential phosphorylation (Figure 4). HEK 293 cells were grown in SILAC media supplemented with heavy or light arginine and lysine for at least five doublings prior to transfection, immunoprecipitation, and SDS-PAGE. Bands containing DCBLD1 or DCBLD2 were excised, subjected to tryptic digestion, and analyzed via LC-MS/MS. Heavy-to-light (H:L) ratios were calculated from monoisotopic peak intensities averaged across elution windows to quantify differential phosphorylation (Table 1, Supplementary Table 4). Three YxxP sites were identified from each molecule, as well as DCBLD2 Tyr715. While using mouse DCBLD1 and human DCBLD2, the numbering used throughout is for human family members.

Taken from the four SILAC pairings not involving kinase inhibitors (unstimulated paired with (i) H_2_O_2_-stimulation, (ii) Fyn co-expression, (iii) Abl co-expression, or (iv) Fyn and Abl co-expression) we detected phosphorylation of Tyr589, Tyr600, and Tyr621 in YxxP sites on DCBLD1 and all were dominantly phosphorylated by Abl. When Abl was co-expressed with DCBLD1 alone or in combination with Fyn, phosphorylation of all three tyrosines were induced between 16 and over 100 fold. None of these sites was observed phosphorylated by Fyn co-expression alone (Table 1). H_2_O_2_-stimulation only induced measurable amounts of Tyr600 phosphorylation on DCBLD1, rendering the use of inhibitors in tandem with H_2_O_2_ only informative for this site. Nonetheless, a twelve-fold decrease in H_2_O_2_-induced phosphorylation was observed at Tyr600 with the Abl-specific inhibitor, further demonstrating that Abl activity is important in the phosphorylation of Tyr600 (Table 1). Notably, each of these phosphorylation sites has complications when quantifying them based on the tryptic peptides that harbor these sites. As regards Tyr589 and Tyr600, each of these sites falls within one tryptic peptide such that singly-and doubly-phosphorylated combinations are possible. Nevertheless, strong Abl-dependent increases were observed for each permutation (Table 1). For phosphorylation of Tyr621, its quantification was complicated given it falls directly N-terminal to an argmine. It is well known that modified amino acids near tryptic cleavage sites can locally decrease trypsin’s proteolytic activity. In this case, we were able to measure Tyr621 phosphorylation only on a larger tryptic peptide, which was not cut after Arg622. Consistent with a phosphorylation-dependent effect on tryptic activity, this larger peptide was not observed without Abl co-expression. Given its absence, we used a local signal-to-noise calculation and found this larger phosphopeptide was increased by more than 100-fold by Abl (Table 1). Furthermore, the fully tryptic unphosphorylated peptide identified in these experiments was reduced by 54% when c-Abl was expressed suggesting the percentage of Tyr621 phosphorylation occupancy was also 54% (Supplementary Table 4).

In SILAC quantification of DCBLD2 YxxP motif phosphorylation, co-expression of Fyn and Abl together generally gave similar increases to H_2_O_2_ stimulation (Table 1). When the kinases were expressed individually with DCBLD2, Fyn was able to induce a more than 25-fold higher level of Tyr565 phosphorylation than Abl and approximately a four-fold higher increase of phosphorylation at Tyr621. On the other hand, Abl induced a higher level of phosphorylation than Fyn at Tyr715 and Tyr750 (17-fold and six-fold respectively). In general, the difference between H_2_O_2_ stimulation alone and in the presence of inhibitors was not strong except in the case where Src-1 was able to reduce phosphorylation of Tyr565 over fifty-fold (Table 1). This was consistent with the approximately 50-fold phosphorylation induction by Fyn at this site. STI571 did reduce phosphorylation of Tyr565, Tyr621, Tyr715, and Tyr750 between four- and ten-fold, but the magnitudes of the changes for Tyr715 and Tyr750 might have been expected to be higher given Abl induced the phosphorylation of these sites 56- and 21-fold respectively (Table 1). However, the expression of c-Abl is likely more aggressive than the endogenous Abl being inhibited by STI571.

Our SILAC comparisons provided an initial baseline survey, but we next turned to label-free (LF) quantification of monoisotopic ion intensities for more rapid replication. This was accompanied by the use of synthetic stable isotope-labeled peptide standards (SL peptides) corresponding to select DCBLD2 tryptic peptides. The chosen peptides harbored phosphorylated or unphosphorylated Tyr565, Tyr715, and Tyr750. We selected these peptides given that, on the one hand tryptic peptides containing phosphorylated Tyr715 and Tyr750 were readily detected, and on the other hand the tryptic peptide containing phosphorylated Tyr565 was more challenging to detect, due in part to the RKKK sequence creating a “ragged” N-terminal side of the tryptic peptide. The SL peptides that were chosen harboring Tyr565 had a single missed cleavage, KTEGTYDLPWDR, as this peptide would likely be a fraction of the fully-tryptic peptide and we found it in a previous study [6]. This SL peptide also allowed us to determine whether the cleavage site preference of trypsin at the N-terminus was condition-dependent or whether accurate changes in phosphorylation could be measured by monitoring just one of the “ragged end” peptides. To do so we compared SL peptide quantifications with LF quantification of peptide ions for KTEGTYDLPWDR and TEGTYDLPWDR individually or using their summed intensities from a single LC-MS/MS run.

**Figure 5:**
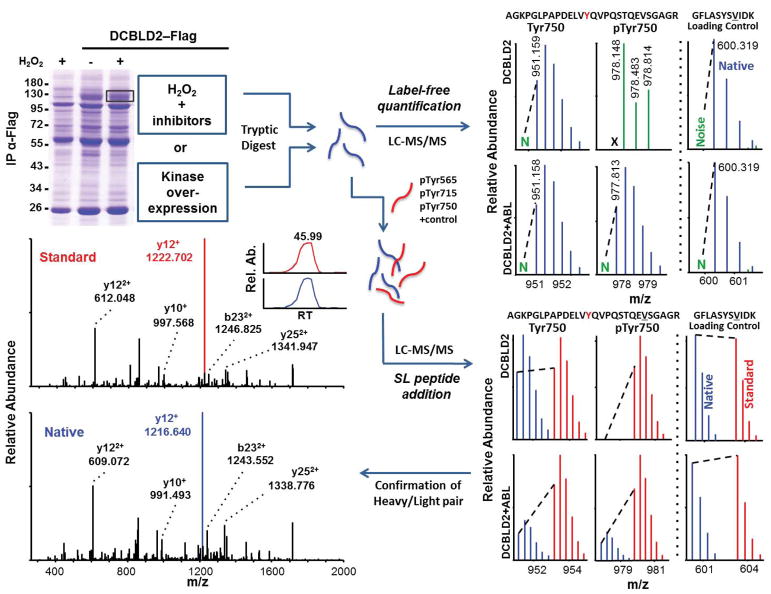
Schematic of sample preparation for quantification using either label-free methods or stable isotope-labeled synthetic reference peptides (SL peptides). Also shown are representative examples of quantification for these two methods by LC-MS/MS. DCBLD2-containing, or control bands were excised from Coomassie-stained gels post-IP from kinase active or inactive/inhibited states. Following in-gel tryptic digestion peptides were analyzed via LC-MS/MS. Label free quantification was achieved by normalizing noise-subtracted monoisotopic peak intensities to loading control peptides for comparison across experimental conditions. SL peptides were spiked into digested native peptides prior to LC-MS/MS. Monoisotopic ratios of native-to-synthetic standard peptides were normalized to reference peptide ratios for comparison across conditions. Heavy and light pairs were confirmed from fragmentation spectra and chromatographic retention time (RT).

Figure 5 displays a schematic of the LF and SL peptide addition methods (see method section for full details). Bands containing the protein of interest were excised from Coomassie-stained gels and digested with trypsin prior to targeted LC-MS/MS scans for phosphorylated and unphosphorylated YxxP-containing peptides. LF quantification was achieved by normalizing the noise-subtracted, monoisotopic intensities of control peptides within DCBLD1 or DCBLD2. These control peptides showed no signs of being modified and therefore served as “loading controls” and allowed direct comparisons of ion intensities across experimental conditions. For quantification using internal standards, the DCBLD2 SL peptides listed above as well as a loading control reference SL peptide were spiked into the native peptide samples just prior to LC-MS/MS analysis. Ratios of native-to-standard monoisotopic peaks of targeted sites were normalized to those of the loading controls for comparison across experimental conditions. Heavy and light pairs were further confirmed through a manual comparison of fragmentation ion spectra (Supplementary Figure 6 and [6]).

**Figure 6:**
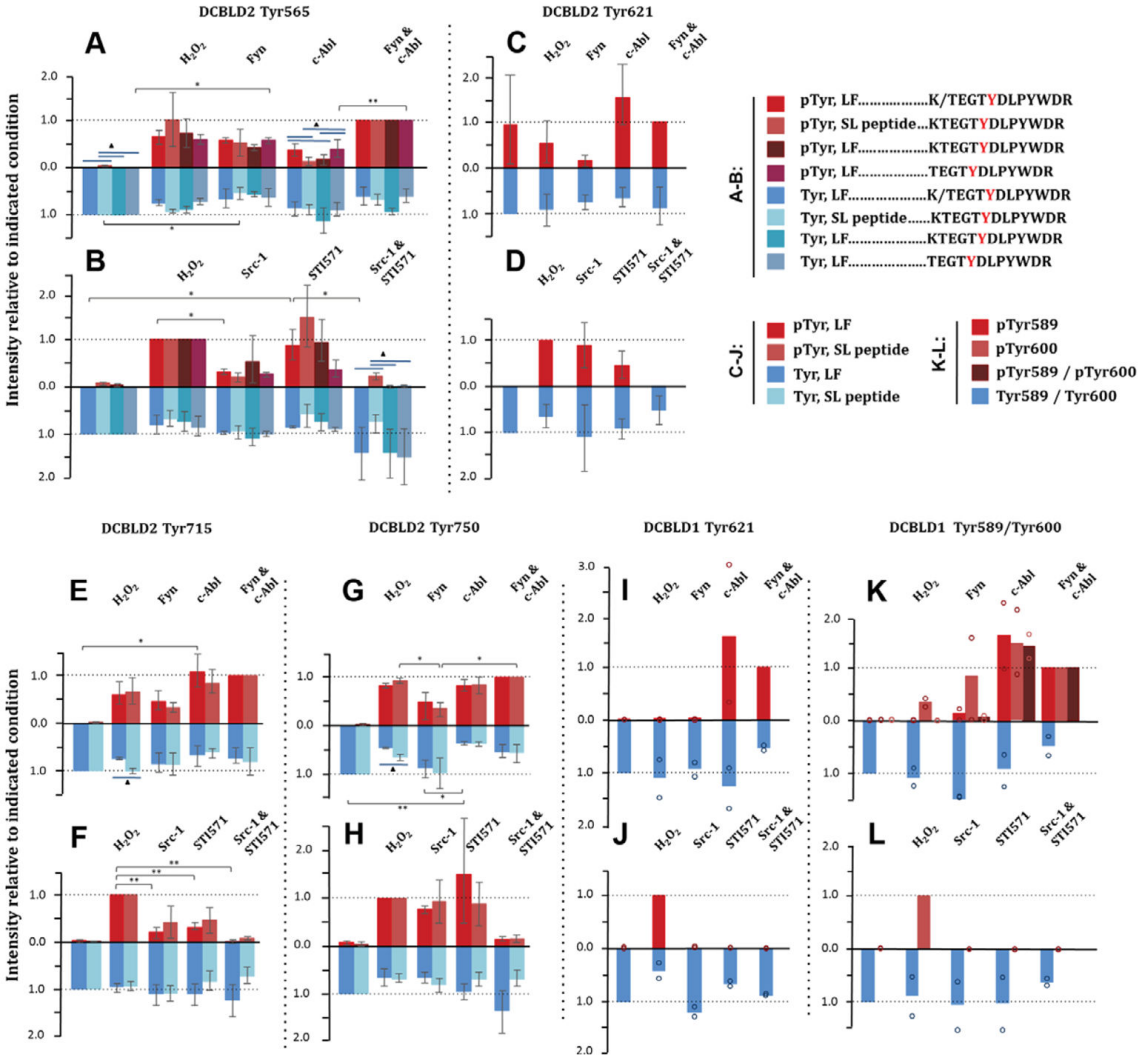
Site-specific quantification of SFK- and Abl-dependent changes in tyrosine phosphorylation of DCBLD1 and DCBLD2 by label-free (LF) or SL peptides. Phosphorylated peptide intensities (red) were normalized to the co-transfection of Fyn and c-Abl in kinase co-expressed conditions, and H_2_O_2_ stimulation for inhibitor treatments. Unphosphorylated peptide ion intensities (blue) were normalized to the unstimulated condition, and the y-axes were inverted to visualize the shift in phosphorylation state. Error bars indicate the standard error of the mean. Column clusters display measurements for the same peptide and condition using different quantification methods. Significance between conditions, indicated by * (*P* < 0.05) or “ (*P* < 0.01), was determined with a one-way ANOVA with a post-hoc Tukey HSD (n=3 biological replicates). Blue bars and triangles indicate significant differences between quantification methods (*P* < 0.05) using either a student’s t-test for a two-method comparison, or the ANOVA/Tukey HSD for more than two methods. The quantification of four DCBLD2 phosphorylation sites are shown in (A–H), including one non-YxxP site. The quantification of three DCBLD1 sites are shown in I–L (n=2 biological replicates and individual data points are provided). Phosphorylation at DCBLD2 Tyr565 was quantified in alternative cleavage states individually, as well as in sum (K/TEGYDLPYWDR) as described in the methods.

Figure 6 displays the LF and SL peptide quantification results for DCBLD2 Tyr565, Tyr621, Tyr715, and Tyr750 (tabulated in Table 2A,B). Phosphopeptides (red) are plotted as a percent relative to the Fyn/c-Abl condition for kinase co-expression experiments or to H_2_O_2_ stimulation when inhibitors were used. Unphosphorylated peptides (blue) were normalized to the unstimulated condition regardless of treatment. Y-axes for unphosphorylated peptides were inverted to best visualize the shift in phosphorylation state of the given peptide. Clusters of bars represent different quantification methods for a given condition. Significant differences between conditions were determined by a one-way ANOVA and Tukey HSD, and are either indicated (* for *P* < 0.05, ** for *P* < 0.01) or are tabulated in Supplementary Table 5A,B. For the method comparison, a one-way ANOVA and Tukey HSD analysis was used in the case of DCBLD2 Tyr565 (>2 methods), while a simple t-test was employed with DCBLD2 Tyr715 and Tyr750 (2 methods). Significant differences among quantification methods are designated by blue bars (triangle for *P* < 0.05). Additional DCBLD2 phospho-tyrosine residues that were identified, but not quantified, are listed in Supplementary Table 6, and include Tyr569, Tyr649, Tyr655, Tyr663 and Tyr677. Tyr569 was not observed in adequate trials to obtain complete quantification of its phosphorylation state, suggesting it is either not highly phosphorylated or it ionizes poorly. The other four sites were on the same tryptic peptide, which was only identified in one LC-MS/MS run.

Significant changes in the phosphorylation state of Tyr565 (Figure 6A,B) were observed in Fyn-active conditions; the abundance of the unphosphorylated peptide decreased relative to the unstimulated condition, with a corresponding increase in the abundance of the phosphopeptide. Further, co-transfection of Fyn and c-Abl resulted in an increased intensity of the phosphopeptide relative to c-Abl alone, which was not significantly different from the unstimulated state, suggesting that Fyn may be the primary kinase of Tyr565. This was corroborated by a decrease in Tyr565 phosphorylation in the presence of Src-1, while STI571 was not able to reduce phosphorylation to the level of the unstimulated condition (Figure 6B). No statistically significant differences were observed in the phosphorylation state of Tyr621 (Figure 6C,D), as there was high variability among conditions when analyzed as a group. However, Fyn alone demonstrated a strong reduction in phosphorylation levels in comparison to Abl. Phosphorylation of the non-YxxP Tyr715 (Figure 6E,F) was induced by both Fyn and Abl, although more strongly by Abl. The inhibitor studies showed that individually the SFK and Abl inhibitors were sufficient to significantly reduce Tyr715 phosphorylation relative to H_2_O_2_ stimulation. Further, application of Src-1 and STI571 together reduced the phosphorylation state beyond that of STI571 alone, suggesting that both Fyn and Abl are involved in the phosphorylation of Tyr715. Tyr750 (Figure 6G,H) demonstrated significantly higher phosphorylation levels by H_2_O_2_ stimulation and by co-expression of Abl compared to co-expression of Fyn. This, together with the ~60% decrease in intensity of the unphosphorylated peptide in the Abl-active condition, indicates that Abl is the primary kinase of Tyr750.

Given that we found Fyn and Abl preferentially phosphorylate Tyr565 and Tyr750 respectively, we asked if the loss of these sites would have important functional consequences on the Fyn- or Abl-induced binding of DCBLD2 to the CRKL-SH2 domain. We made use of tyrosine-to-phenylalanine mutant DCBLD2 expression constructs (Figure 2A) [6], where a DCBLD2-Y1F construct harbored a Tyr750Phe mutation, and a DCBLD2-Y3F mutant harbored Tyr565Phe, Tyr732Phe and Tyr750Phe. The DCBLD2-Y7F construct had tyrosine-to-phenylalanine mutations at all seven intracellular YxxP motifs. Analyses using these constructs, in tandem with the phospho-tyrosine quantification by mass spectrometry, were helpful in specifying which tyrosine residues are most important in the Fyn- or Abl-mediated DCBLD2-CRKL-SH2 interaction. The DCBLD2-Y1F construct showed little reduction in binding induced by either kinase (Supplementary Figure 5A,B) demonstrating that phosphorylation at Tyr750 was not strictly required for the DCBLD2-CRKL interaction induced by Fyn or Abl. While DCBLD2-Y3F was induced to bind upon stimulation by each kinase, the interaction with DCBLD2-Y3F was reduced in comparison to the DCBLD2-Y1F mutant or WT DCBLD2. As Tyr732 was not found to be phosphorylated by Fyn or Abl by LC-MS/MS, these analyses suggest that together Tyr565 and Tyr750 contribute to Fyn- and Abl-induced binding of DCBLD2 to the CRKL-SH2 domain. As predicted, DCBLD2-Y7F showed complete loss of Fyn- and Abl-induced binding to the CRKL-SH2 domain. The graded loss of CRKL-SH2 binding between DCBLD2 WT, Y1F, Y3F and Y7F denotes the role of multi-site phosphorylation in this interaction and predicts a role for avidity in the strength of the interaction.

For DCBLD1 three phosphorylation sites were identified: Tyr621, Tyr589, and Tyr600. The latter two sites were on the same tryptic peptide. Phosphorylation of Tyr621 was quantified from the sum of monoisotopic intensities of two cleavage patterns surrounding this site. As with the SILAC experiments described above, a decreased preference for cleavage after Arg622 was readily observed when phosphorylation was found at Tyr621. However, we did identify the presence of the phosphorylated fully cleaved ion and unphosphorylated ion with one missed cleavage using targeted precursor ion scans. Raw intensities of the unphosphorylated and phosphorylated fully cleaved peptides were summed with their uncleaved counterparts in order to obtain a clear picture of phosphorylation at this site. While the two trials completed did not permit statistical analysis, Fyn did not appear to be the primary Tyr621 kinase as phosphorylation levels by co-expression of Fyn or by H_2_O_2_ stimulation were comparably weak. On the other hand, co-expression of Abl alone or in combination with Fyn led to strong increases in phosphorylation, well above that of H_2_O_2_ stimulation (Figure 6I). While treatment with Src-1 and STI571 resulted in similar decreases in pTyr621 (Figure 6J), the possibility of Abl activation downstream of SFKs in the oxidative stress response suggests that the reduction in signal upon Src-1 treatment could be due to a reduction in Abl activity. Together with the kinase expression data, this suggests that Abl is the primary kinase of Tyr621. The peptide harboring both Tyr589 and Tyr600 was quantified in various states of phosphorylation; singly phosphorylated peptides were individually quantified at each YxxP site, and separately from the doubly phosphorylated peptide. Fyn was able to induce phosphorylation at Tyr600 only, while c-Abl co-expression resulted in high signal intensity of all three phosphorylation states. Only H_2_O_2_ stimulation was capable of bringing pTyr600-containing peptides to sufficient concentrations to analyze via LC-MS/MS, complicating quantification with inhibitor treatment. The complete loss of signal of pTyr600 upon inhibitor treatment, similar to pTyr621, suggests that the activity of Abl is necessary for phosphorylation at this site.

## DISCUSSION

**Figure 7:**
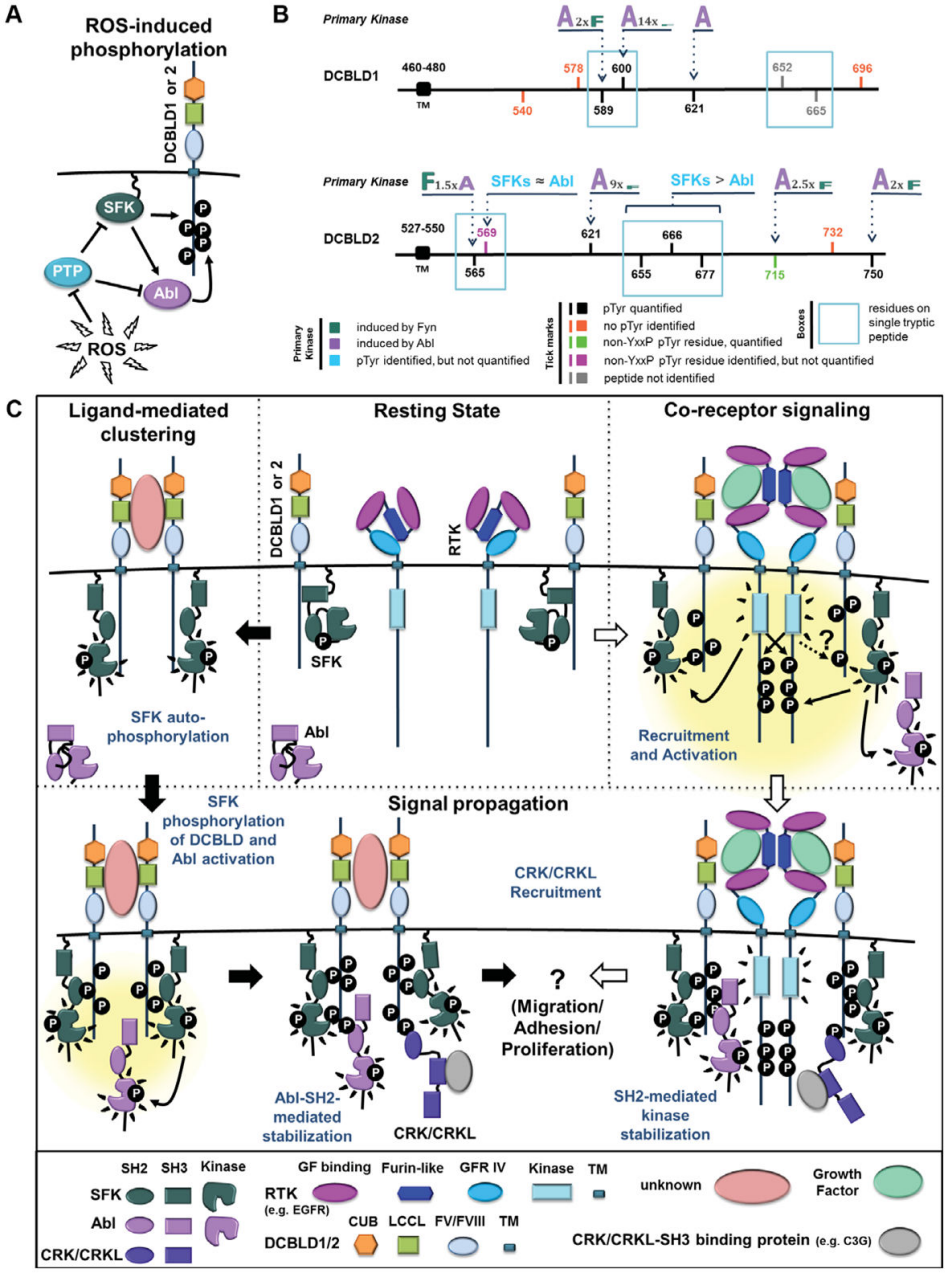
Mechanisms of SFK-/Abl-driven phosphorylation of DCBLD proteins and CRKL recruitment. (A) ROS-driven phosphorylation model. Generation of ROS following H_2_O_2_ treatment of cells in culture inhibits tyrosine phosphatases, thereby activating SFKs, which in turn activate Abl. Together, SFKs and Abl contribute to the phosphorylation of DCBLD family members. The same effect was achieved by co-expression Fyn or c-Abl with DCBLD1/2 to determine the relative contribution of each kinase. (B) Summary depicting the relative site-specific targeting of Abl and Fyn in the tyrosine phosphorylation of DCBLD1 and DCBLD2. Tick marks within the intracellular domains of DCBLD1 and DCBLD2 indicate tyrosines within YxxP motifs or non-YxxP tyrosines that were found phosphorylated. The relative targeting of each quantified site is demonstrated by the size of the letter: “A” for Abl or “F” for Fyn. The magnitude of the relative difference is also indicated for each site. (C) Signaling model for SFK-/Abl-driven CRKL recruitment to DCBLD family scaffolds. In the ligand-mediated clustering model, extracellular ligand binding clusters DCBLD1/2 and membrane-anchored SFKs, leading to SFK auto-phosphorylation and activation of kinase activity. Active SFKs then phosphorylate clustered DCBLD1/2 on intracellular tyrosines, providing a docking site for SFK-SH2 domains to stabilize the open, active conformation [6]. Active SFKs also phosphorylate Abl Tyr412, activating Abl kinase activity. Abl-SH2-mediated stabilization leads to increased levels of DCBLD pYxxP, recruiting CRK/CRKL to bind the DCBLD scaffold via the SH2 domain. CRK/CRKL-SH3-bound cargo (e.g. C3G) is brought to the membrane through this interaction. In the co-receptor signaling model, DCBLD1/2 act as co-receptors with growth factor RTKs (e.g. EGFR). Upon extracellular growth factor (GF) binding, RTK auto-phosphorylate and activate membrane-anchored SFKs. SFKs phosphorylate DCBLD1/2 co-receptors and Abl, leading to kinase SH2-mediated stabilization, signal propagation, CRK/CRKL recruitment, and ultimately to CRK/CRKL-related processes such as cell migration, adhesion, or proliferation. Included under the figure is a key indicating the color-coding of protein domains.

This study characterized the intracellular tyrosine phosphorylation of the DCBLD family of transmembrane orphan receptors summarized in the model and data shown in Figure 7. Our data revealed site-specific regulation of phosphorylation by Abl and Fyn, showing that phosphorylation at multiple tyrosines induces DCBLD proteins to bind to the CRKL-SH2 domain. Our results are summarized in Figure 7B and in general find that specific DCBLD1 YxxP sites (Tyr589, Tyr600 and Tyr621) are strongly targeted by Abl while DCBLD2 YxxP sites are in general targets of both Abl and Fyn. However, Abl demonstrated preferences for DCBLD2 Tyr750 and Tyr715 and Fyn had a preference for Tyr565 and likely Tyr655, Tyr666 and Tyr677. We used our quantitative MS data to calculate the relative induction of distinct phosphorylation sites of DCBLD1 and DCBLD2 by Abl and Fyn (Figure 7B). These ratios describe an approximate fold change of molecules phosphorylated at a given site under the indicated cellular states.

To our knowledge, this is the first side-by-side comparison of label free methods to internal standard addition and SILAC in targeted quantification of PTMs. SL peptide addition holds an advantage over other methods, as the user is provided with a strong MS2 fingerprint to aid in the identification of low abundance endogenous peptides harboring PTMs. When relying on a spectral matching algorithm alone, fragmentation spectra of low abundance peptides may be lost through the peptide filtration process (e.g. due to low cross-correlation scores using SEQUEST). In the simple identification or verification of site-specific PTMs that are present in low abundances, standard addition would present an advantage over SILAC or LF methods. However, in the quantification of multiple sites across a variety of conditions as described here, SL peptides can artificially inflate native-to-standard ratios in cases where no modified peptide is identified. For such quantitative comparisons, the LF method is advantageous, as it provides the more accurate representation as minimal-to-no signal above the level of noise. Overall, LF and SL quantification yielded similar results, demonstrating the merits of LF quantification. In low-complexity samples, such as peptides derived from an immunoprecipitated band as used here, the known challenges of ion suppression in LF quantification [28] are minimized. While SILAC quantification has the advantage of providing a side-by-side comparison of all peptides across 2–3 treatments, we found LF analysis was less cumbersome and less costly given we were quantifying many samples from several treatments. However, in large-scale quantification discovery analyses of PTMs across only a few experimental conditions, SILAC would be a strong choice.

Additional merits of LF quantification over SL peptides include the ability to quantify different enzymatic cleavage patterns. Using the quantification of DCBLD2 pTyr565 as an example, the summed raw intensities of KTEGTYDLPYWDR and TEGTYDLPYWDR were not statistically different than the most abundant fully cleaved peptide. This demonstrates that quantification of the most abundant cleavage pattern will generally yield an accurate measurement in cases where the PTM falls far from cleavage sites. However, PTMs close to the enzyme cleavage site can reduce the specificity of the enzyme for that site, as was observed with the tryptic cleavage of DCBLD1 Tyr621, and it may be necessary to sum the intensities of fully cleaved and miss-cleaved peptides. Using LF methods in such cases allows the investigator flexibility in quantification post-LC-MS/MS. These findings demonstrate the importance in choice of quantification method, and the need to consider the peptide harboring the PTM when determining which method is most appropriate.

Future work will characterize whether phosphorylation of any individual tyrosine residue reported here is dependent upon phosphorylation of other sites. As we observed with H_2_O_2_ stimulation in DCBLD2-transfected cells, endogenous Abl activity was maintained above that in untransfected cells stimulated with H_2_O_2_ by a possible binding interaction between the Abl-SH2 domain and at least one of the DCBLD2 YxxP motifs. This activity was reduced to baseline levels observed in the mock when all seven YxxP sites were mutated to FxxP (Supplementary Figure 2B). Others have demonstrated that Abl activity requires binding to phosphotyrosine-containing proteins through the Abl-SH2 domain [29–31]. Further, it is hypothesized that phosphoprotein binding to the SH2 domain is the mechanism of activation for c-Abl through RTKs, as has been shown with EphB2 and Trk [32, 33]. Our reported dependence of Abl activity and SH2 domain binding on DCBLD2 pYxxP sites suggests that Abl-directed phosphorylation of DCBLD2 requires priming of the Abl-SH2 binding site. If specific YxxP sites are essential for Abl-SH2 binding, phosphorylation of other sites could be dependent upon phosphorylation of the Abl-SH2 binding site. It will also be important to determine whether proteins in addition to CRKL are induced to bind differentially upon SFK- or Abl-induced phosphorylation.

Our working DCBLD signaling model is summarized in Figure 7C. We have previously demonstrated antibody-induced phosphorylation of DCBLD2, which suggests a ligand-mediated clustering mechanism (Figure 7C) [6]. We hypothesize that clustering leads to SFK activation at the membrane and subsequent Abl activation, either through direct phosphorylation by SFKs or Abl-SH2 binding to SFK-directed pYxxP [34–37], leading to increased levels of DCBLD1/2 tyrosine phosphorylation and recruitment of CRK/CRKL. Future work will strive to identify an extracellular ligand that induces intracellular phosphorylation and CRK/CRKL recruitment. In an alternative signal transduction mechanism, others have demonstrated growth factor-induced DCBLD2 phosphorylation [7] and DCBLD2-RTK complex formation [38]. We present a co-receptor signaling mechanism (Figure 7C) in which growth factor binding to EGFR, a representative growth factor receptor, induces SFK and Abl activation, as well as DCBLD1/2 phosphorylation and CRK/CRKL recruitment. It is unknown whether DCBLD1/2 phosphorylation occurs directly via RTK activity, or indirectly through the activation of non-receptor tyrosine kinases.

While the biological roles of DCBLD family tyrosine phosphorylation sites remain to be elucidated, their strong conservation across vertebrates implies they mediate important signaling events. Our characterization here of the overlapping roles of SFKs and Abl in DCBLD family phosphorylation and how this multisite phosphorylation induces CRKL binding provides a framework for further cellular biochemical studies of this new receptor family.

## Supplementary Material



## Figures and Tables

**Table 1:** SILAC identifies tyrosine phosphorylation sites on DCBLD1 and DCBLD2 differentially regulated by SFKs and Abl. As indicated, heavy conditions either possessed a co-expressed kinase when paired with a light unstimulated condition, or H_2_O_2_ stimulation alone when paired with a light inhibitor treatment followed by H_2_O_2_ stimulation. The identified phosphotyrosine (Y#)-containing peptides and the number of the phosphorylated residue (human numbering are indicated on the left. For each phosphopeptide and each condition the H:L ratios (fold change) are listed.

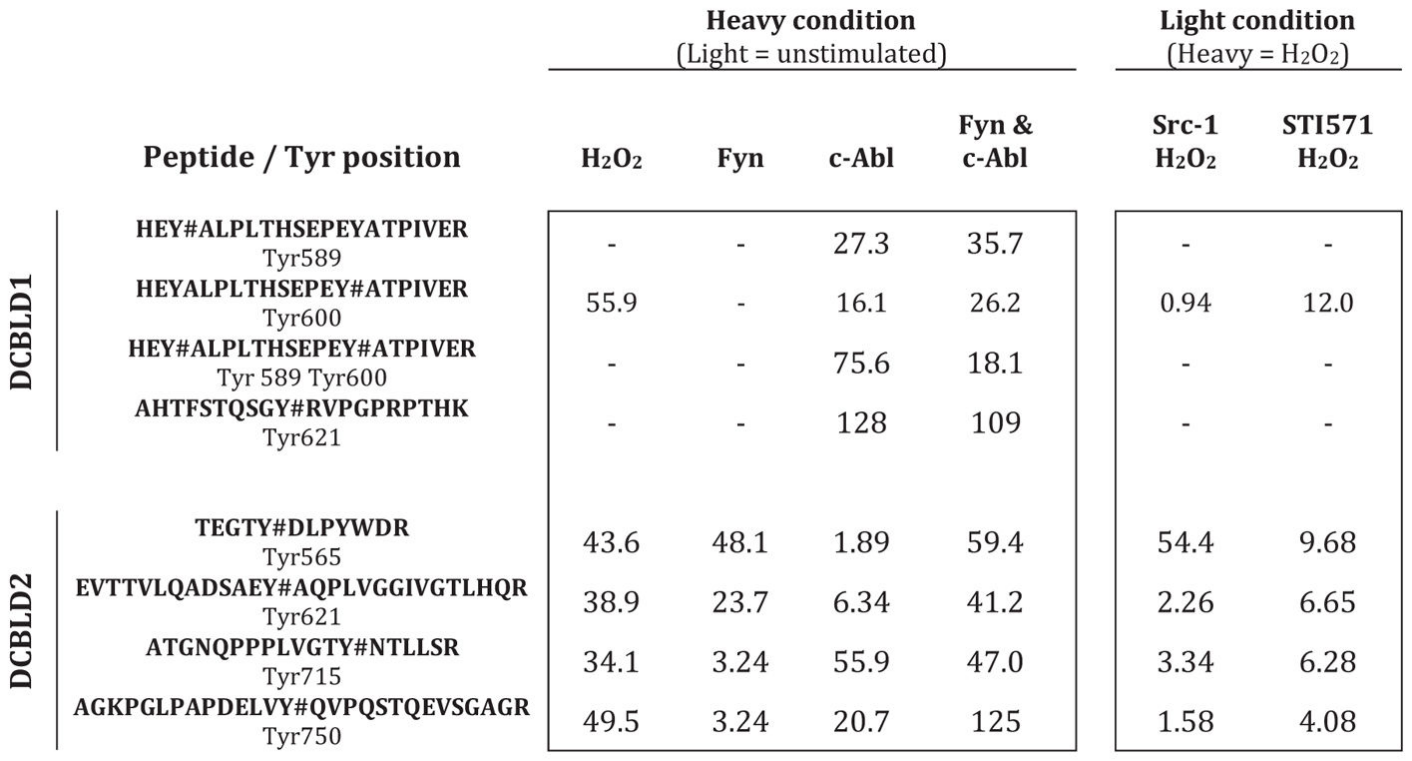

**Table 2:** Quantification of DCBLD1 and DCBLD2 tyrosine phosphorylation with label-free (**LF**) and SL peptides (*SL*). Tabulated averages of normalized phosphorylated peptide and unphosphorylated peptide ion intensities as graphed in Figure 6. Phosphorylated peptide ion intensities were normalized (boxed values) either to dual co-expression of Fyn and c-Abl (in kinase co-expression studies) or H_2_O_2_ stimulation (in inhibitor treatment studies). Unphosphorylated peptide ion intensities were normalized to the unstimulated condition in all cases. Tabulated values for each DCBLD family member and the sets of conditions are as follows: kinase co-expression studies with (A) DCBLD2 and (B) DCBLD1 and inhibitor studies with (C) DCBLD2 and (D) DCBLD1.

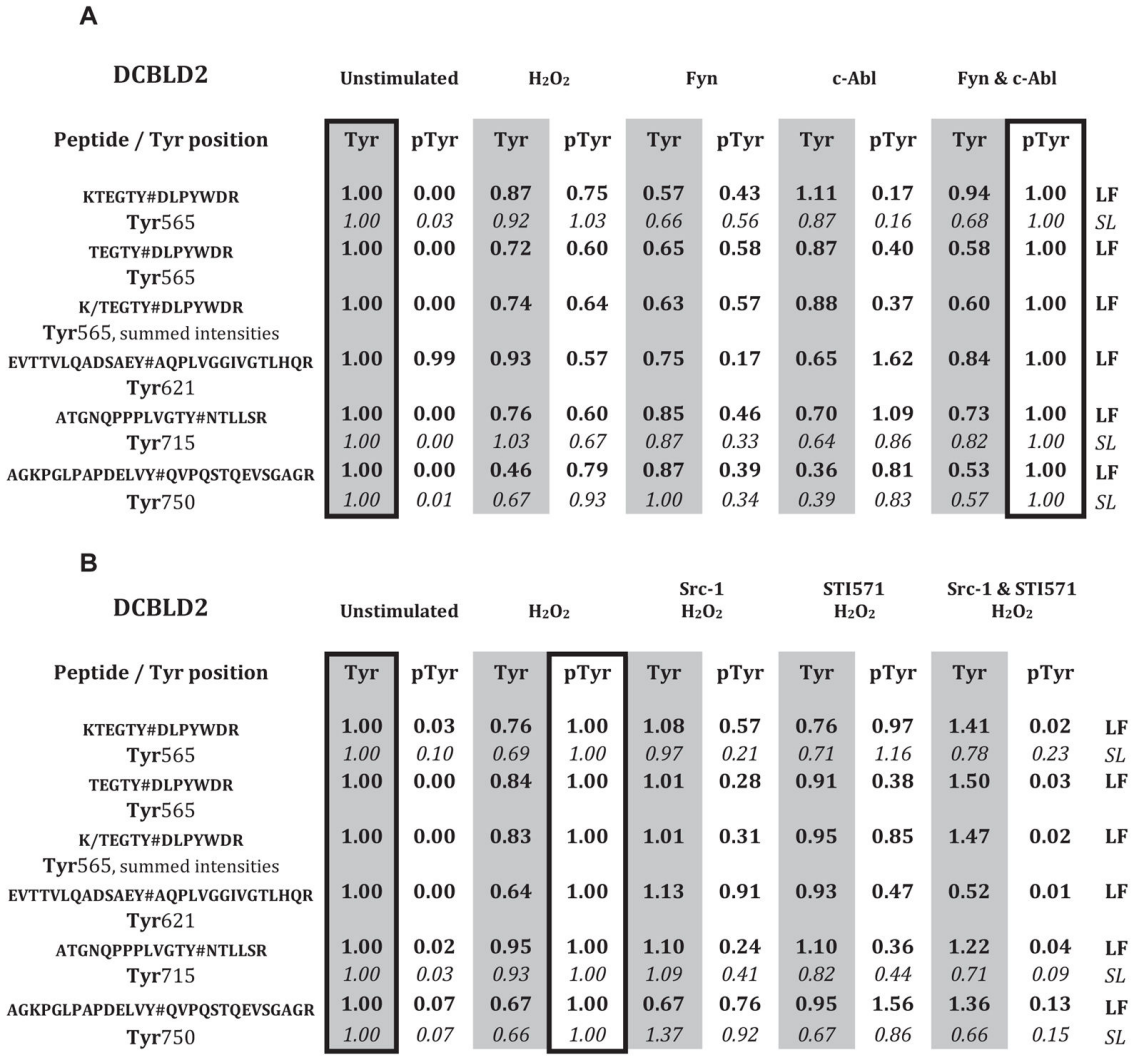
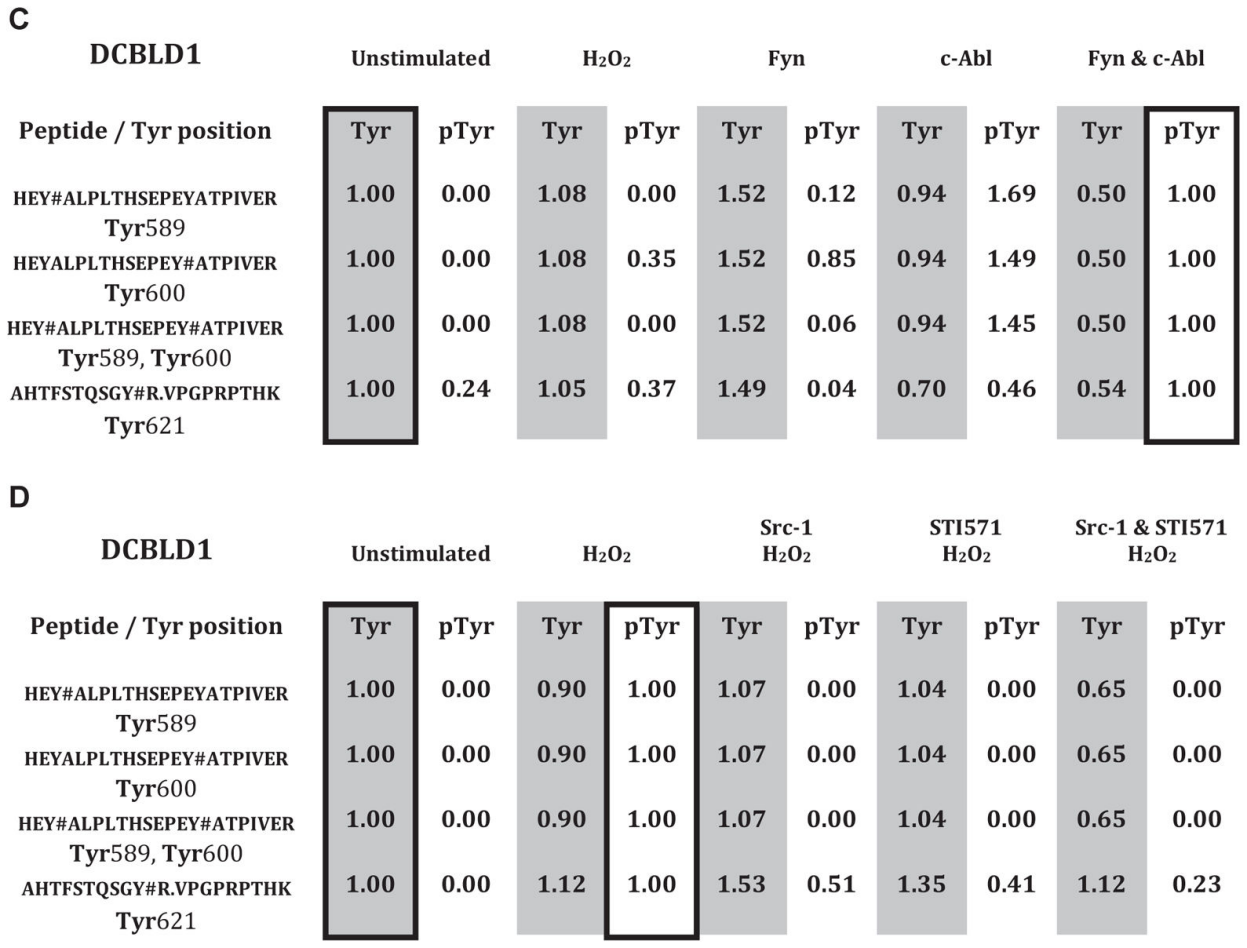

## ABBREVIATIONS USED

Ab: antibody
Akt: Ak strain thymoma
BCLB: brain complex lysis buffer
BLB: bacterial lysis buffer
BSA: bovine serum albumin
CID: collision-induced dissociation
CIP: calf intestinal phosphatase
DCBLD1/2: discoidin, CUB, and LCCL domain-containing 1/2
DMEM: Dulbecco’s modified eagle’s medium
DMSO: dimethyl sulfoxide
ECL: enhanced chemiluminescence
EGF: Epidermal Growth Factor
ESDN: endothelial smooth muscle cell-derived neuropilin-like
FA: formic acid
FWHM: full width half maximum
GST: Glutathione S-transferase
HEK: human embryonic kidney
HPLC: high performance liquid chromatography
HRP: horseradish peroxidase
IP: immunoprecipitation
IPTG: isopropyl β-D-1-thiogalactopyranoside
IR: Insulin Receptor
KD: kinase-dead
LB: Luria broth
LF: label-free
LC-MS/MS: liquid chromatography tandem mass spectrometry
MAPK: Mitogen Activated Protein Kinase
PD: pull-down
PDGF: Platelet-Derived Growth Factor
PMSF: phenylmethylsulfonyl fluoride
PTM: post-translational modifications
RTK: receptor tyrosine kinase
SDS-PAGE: sodium dodecyl sulfate polyacrylamide gel electrophoresis
SFK: Src family kinase
SH2: Src homology 2
SH3: Src homology 3
SILAC: stable isotope labeling of amino acids in cell culture
SL: synthetic stable isotope-labeled peptide standards
TBS-T: Tris-buffered salin–polysorbate 20
TM: transmembrane
TRAF6: TNF receptor-associated factor 6
VEGF: Vascular Endothelial Growth Factor
WCE: whole cell extract
WT: wild-type
XIC: extracted ion chromatogram
