# Supplementary material for: Dynamic multi-site phosphorylation by Fyn and Abl drives the interaction between CRKL and the novel scaffolding receptors DCBLD1 and DCBLD2
Source: Biochem J. Author manuscript; Available in PMC 2018 Nov 21. (PMC6029619; doi:10.1042/BCJ20170615)
Supplement: Supplementary file 1 [file NIHMS976803-supplement-supplement_1.pdf]

Supplementary Figure 1. YxxP motifs in human DCBLD1 are required for H_2O_2-induced binding to the CRKL-SH2 domain. GST-CRKL-SH2 pull-down assays comparing human DCBLD1-Flag WT and human DCBLD1-Flag Y8F. HEK 293 cells transfected with the indicated constructs were either left untreated or stimulated with H_2O_2 for 20 minutes prior to lysis and pulldown assays from the clarified extracts. The immunoblot of the pulldown assays was first stained with Ponceau to visualize levels of the GST-CRKL-SH2 domain. This was followed by blotting with anti-Flag. Levels of the DCBLD1-Flag proteins were determined by immunoprecipitation and immunoblotting of the immune complexes following their separation by SDS-PAGE. Whole cell extracts were subjected to SDS-PAGE and immunoblotting with anti-phosphotyrosine to verify the effect of H_2O_2 stimulation.

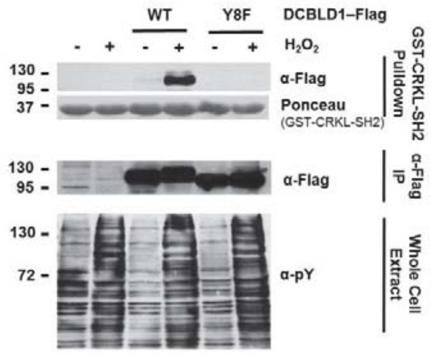

Supplementary Figure 2. (A) Src-1 and STI571 titrations to determine concentrations of SFK and Abl inhibitors necessary to disrupt the DCBLD2-CRKL-SH2 interaction. GST-CRKL-SH2 pulldown assays were performed from DCBLD2-transfected HEK 293 lysates treated with 0.5, 1, or 2 μM Src-1 or 5, 10, or 20 μM STI571 for 15 min, followed by an additional 15 min in 8.8 mM H_2O_2 treatment prior to lysis. Neither inhibitor was sufficient to abolish the interaction alone, however, 2 μM Src-1 and 20 μM STI571 were deemed appropriate to prevent potential off-target effects at higher concentrations. (B) Tyrosine residues in YxxP motifs are required for the increased Abl (and SFK) activity observed in WT DCBLD2-transfected HEK 293 cells treated with H_2O_2. Compare differences observed between pAbl and pSrc blots in lanes 1-3 of panels A and B. In B, HEK 293 cells expressing DCBLD2-Y7F-Flag were treated with H_2O_2 alone or with inhibitors and H_2O_2 prior to lysis as described for A. Whole cell extracts were subjected to SDS-PAGE and proteins were immunoblotted with the indicated antibodies. c) DCBLD2 Tyr phosphorylation in YxxP motifs are required for the ABL-SH2 domain to bind. DCBLD2-WT-Flag and DCBLD2-Y7F-Flag were immunoprecipitated from transfected HEK 293 cells treated with or without H_2O_2. Immune complexes were then incubated with the GST-ABL-SH2 fusion protein. While some background binding was observed in all lanes of the anti-GST blot, significantly higher levels of the GST-ABL-SH2 protein bound to DCBLD2-WT when HEK 293 cells were treated with H_2O_2 prior to lysis, suggesting the Tyr residues in YxxP motifs are required to the DCBLD2-ABL-SH2 interaction. The "*" on the panel showing Flag reactivity indicates the signal from a heavy-chain dimer of the antibody in the IP.

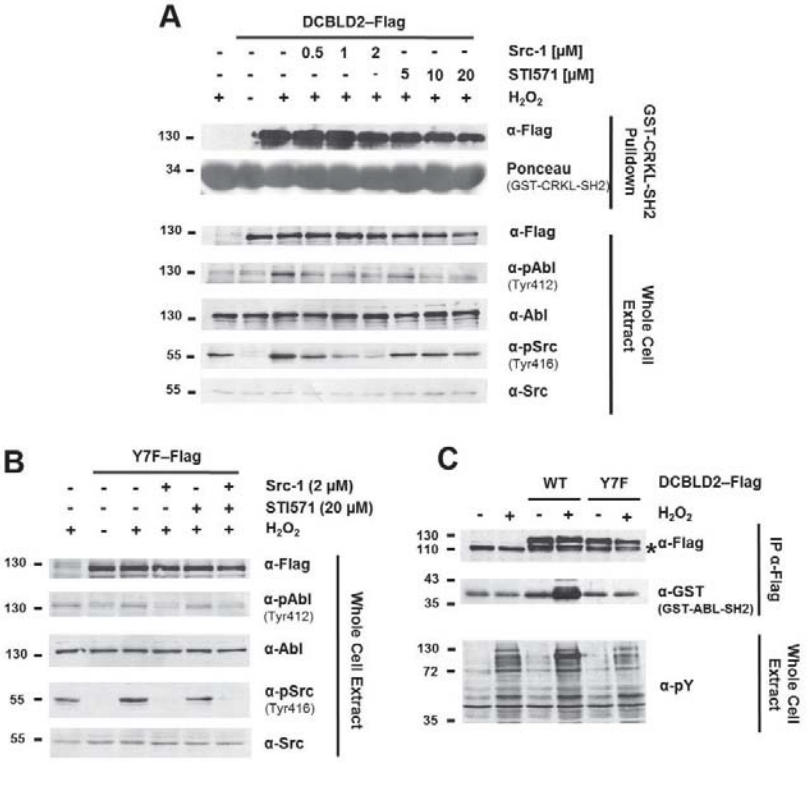

Supplementary Figure 3. Workflow for LC-MS/MS quantification of regulated tyrosine phosphorylation sites on DCBLD1 and DCBLD2. Flag-tagged DCBLD proteins were expressed in HEK 293 cells under two general types of conditions: (i) cells were left unstimulated, stimulated with H_2O_2, or pre-treated with SFK and/or Abl inhibitors prior to H_2O_2 stimulation; and (ii) DCBLD proteins were co-expressed with Fyn and/or Abl. DCBLD proteins were immunoprecipitated from cell extracts and the immune complexes were separated by SDS-PAGE. DCBLD proteins were subjected to in-gel tryptic digestion and phosphorylation of intracellular YxxP sites were monitored with targeted LC-MS/MS of tryptic phosphopeptides. The DCBLD2 peptide harboring the non-YxxP Tyr715 was also targeted in our analysis. Quantification was achieved by three distinct methods for comparison (SILAC, label-free, and the addition of stable isotope-labeled peptide standards).

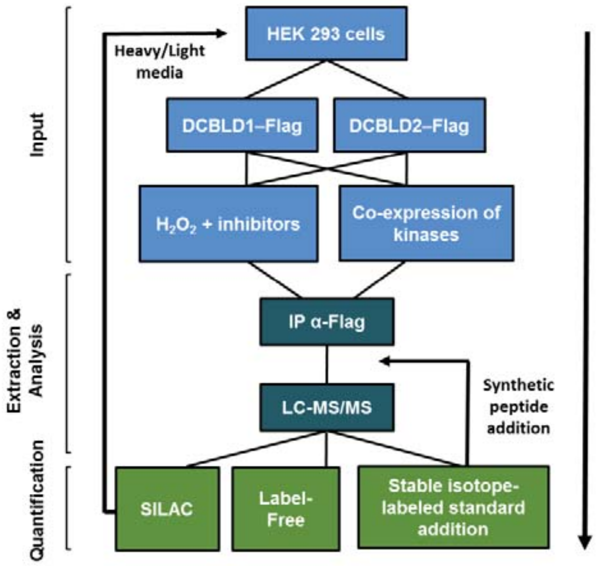

Supplementary Figure 4. Amino acid sequences, tryptic cleavage sites, and LC-MS/MS coverage maps of (A) mouse DCBLD1 and (B) human DCBLD2. Arg and Lys residues in red indicate anticipated cleavage sites within each protein during the proteolytic digestion. Underlined portions of each sequence denote coverage observed via LC-MS/MS.

A

MGTGAGGPSVLALLFAVCAPLRLQAEELGDGCGHIVTSQDSGTMTSKNYPGTYPNYTVCEKIITVPKGKRLILRLGD
LNIESKTCASDYLLFSSATDQYGPYCGSWAVPKELRLNSNEVTVLFKSGSHISGRGFLLTYASSDHPDLITCLERGS
HYFEEKYSKFCPAGCRDIAGDISGNTKDGYRDTSLLCKAAIHAGIITDELGGHINLLQSKGISHYEGLLANGVLSRH
GSLSEKRFLFTTPGMNITTVAIPSVIFIALLLTGMGIFAICRKRKKKGNPYVSADAQKTGCWKQIKYPFARHQSTEF
TISYDNEKEMTQKLDLITSDMADYQQPLMIGTGTVARKGSTFRPMDTDTEEVRVNTEASGHYDCPHRPGRHEYALPL
THSEPEYATPIVERHLLRAHTFSTQSGYRVPGPRPTHKHSHSSGGFPPATGATQVESYQRPASPKPVGGGYDKPAAS
SFLDSRDPASQSQMTSGGDDGYSAPRNGLAPLNQTAMTALL

B

MASRAVVRARRCPQCPQVRAAAAAPAWAALPLSRSLPPCSNSSSFSMPLFLLLLLVLLLLLEDAGAQQGDGCGHTVL
GPESGTLTSINYPQTYPNSTVCEWEIRVKMGERVRIKFGDFDIEDSDSCHFNYLRIYNGIGVSRTEIGKYCGLGLQM
NHSIESKGNEITLLFMSGIHVSGRGFLASYSVIDKQDLITCLDTASNFLEPEFSKYCPAGCLLPFAEISGTIPHGYR
DSSPLCMAGVHAGVVSNTLGGQISVVISKGIPYYESSLANNVTSVVGHLSTSLFTFKTSGCYGTLGMESGVIADPQI
TASSVLEWTDHTGQENSWKPKKARLKKPGPPWAAFATDEYQWLQIDLNKEKKITGIITTGSTMVEHNYYVSAYRILY
SDDGQKWTVYREPGVEQDKIFQGNKDYHQDVRNNFLPPIIARFIRVNPTQWQQKIAMKMELLGCQFIPKGRPPKLTQ
PPPPRNSNDLKNTTAPPKIAKGRAPKFTQPLQPRSSNEFPAQTEQTTASPDIRNTTVTPNVTKDVALAAVLVPVLVM
VLTTLILILVCAWHWRNRKKKTEGTYDLPYWDRAGFYLMVSLACRHNEGWWKGMKQFLPAKAVDHEETPVRYSSSEV
NHLSPREVTTVLQADSAEYAQPLVGGIVGTLHQRSTFKPEEGKEAGYADLDPYNSPGQEVYHAYAEPLPITGPEYAT
PIIMDMSGHPTTSVGQPSTSTFKATGNQPPPLVGTYNTLLSRTDSCSSAQAQYDTPKAGKPGLPAPDELVYQVPQST
QEVSGAGRDGECDVFKEIL

Supplementary Figure 5. Several DCBLD2 YxxP Tyr residues contribute to the ability of Fyn and Abl to induce DCBLD2 to bind to CRKL-SH2 domain. DCBLD2 WT as well as tyrosine-to-phenylalanine point mutants (Y1F, Y3F, and Y7F) were expressed in cells with or without co-expression of Fyn (A) or Abl (B). All DCBLD2 constructs have both Myc and Flag tags at their C-terminus. Cell extracts were subject to pulldown assays with GST-CRKL-SH2 or immunoblotting with the indicated antibodies. As the Abl kinase we are using has a Flag-tag, anti-Myc was used in the immunoblots.

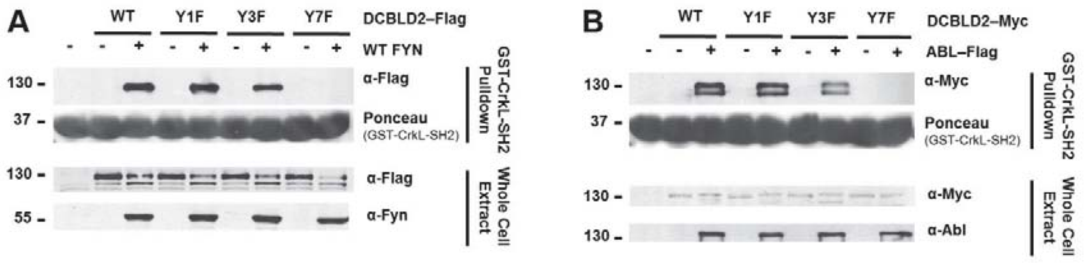

Supplementary Figure 6. Low energy CID fragmentation spectra of DCBLD1 and DCBLD2 tryptic peptide ions harboring differentially regulated phosphorylated and unphosphorylated tyrosine residues. “Y#” denotes phosphorylated tyrosines. Spectra were acquired in a linear ion trap mass spectrometer. Parent ion masses and charge states are tabulated in Supplementary Table 1. Included are MS/MS spectra of unphosphorylated, singly phosphorylated, and multiply phosphorylated peptides harboring more than one tyrosine. MS/MS spectra of phosphorylated and unphosphorylated tryptic peptide ions harboring (A-D) DCBLD1 Tyr589 and Tyr600 (E-J) DCBLD2 Tyr565 and Tyr569, (K-Q) DCBLD2 Tyr649, Tyr655, Tyr663, Tyr666 and Tyr677.

A

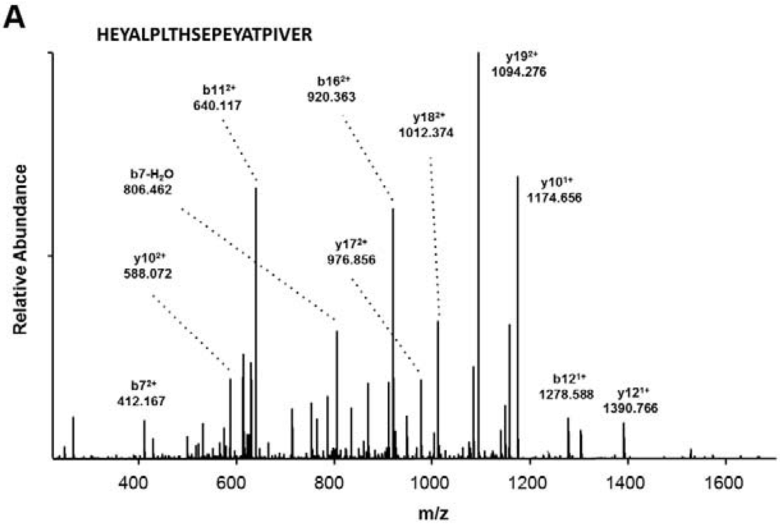

B

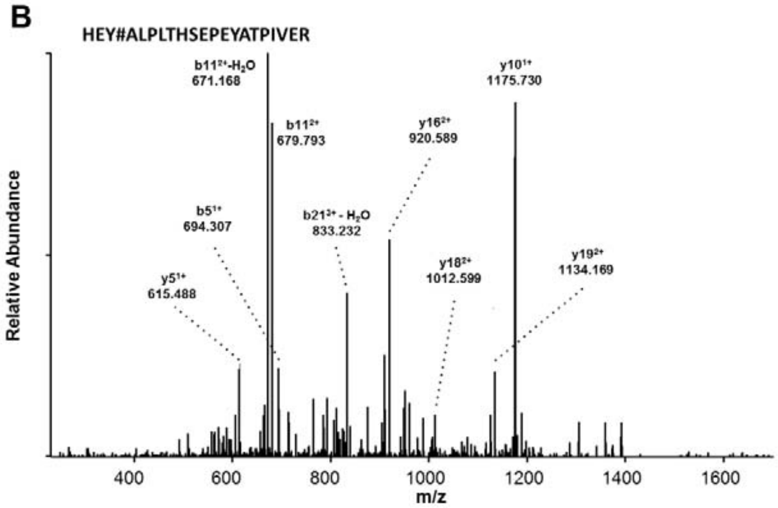

C

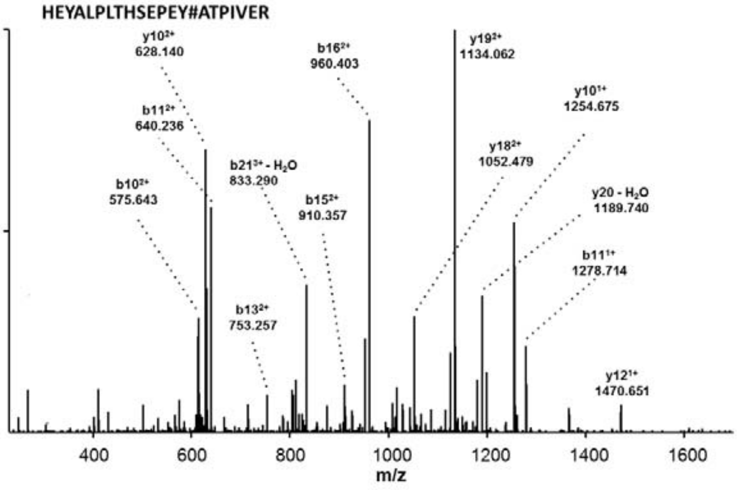
HEYALPLTHSEPEY#ATPIVER
y10²⁺ 628.140
b11²⁺ 640.236
b10²⁺ 575.643
b21³⁺ - H₂O 833.290
b16²⁺ 960.403
b15²⁺ 910.357
b13²⁺ 753.257
y19²⁺ 1134.062
y18²⁺ 1052.479
y10¹⁺ 1254.675
y20 - H₂O 1189.740
b11¹⁺ 1278.714
y12¹⁺ 1470.651
Relative Abundance
m/z
400
600
800
1000
1200
1400
1600

D

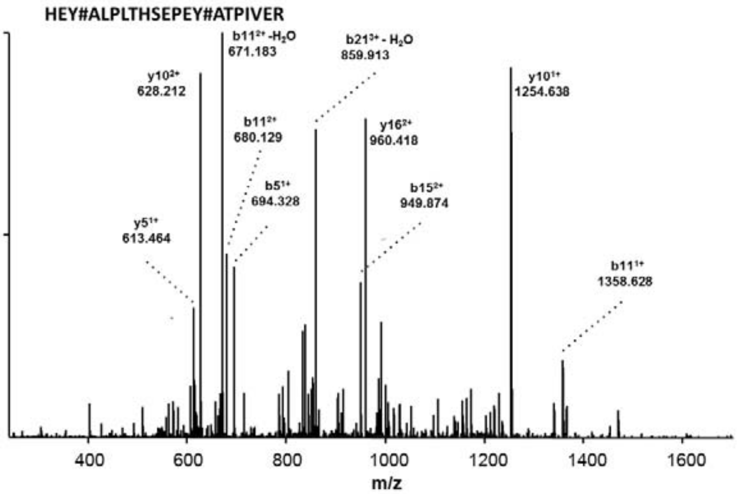
HEY#ALPLTHSEPEY#ATPIVER
y10²⁺ 628.212
b11²⁺ -H₂O 671.183
b11²⁺ 680.129
b5¹⁺ 694.328
y5¹⁺ 613.464
b21³⁺ - H₂O 859.913
y16²⁺ 960.418
b15²⁺ 949.874
y10¹⁺ 1254.638
b11¹⁺ 1358.628
Relative Abundance
m/z
400
600
800
1000
1200
1400
1600

E

TEGTYDLPYWDR

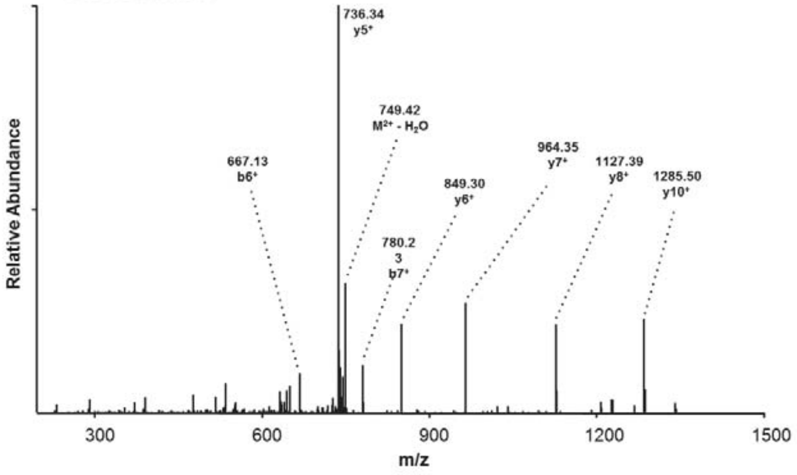

F

TEGTY#DLPYWDR

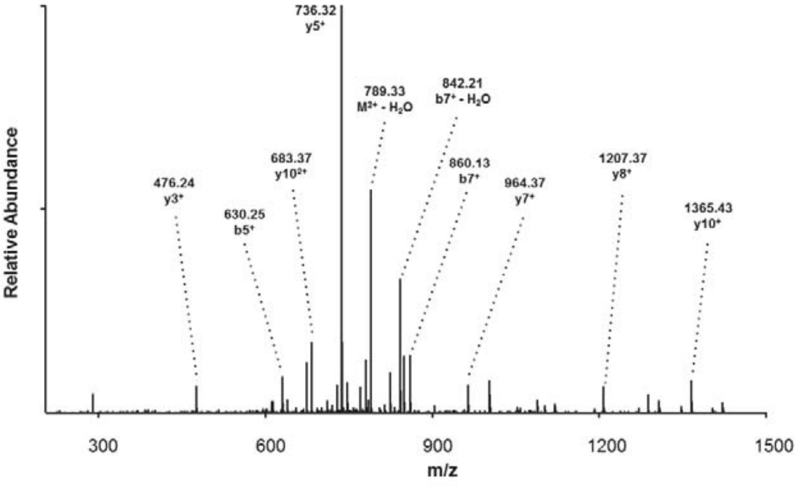

G

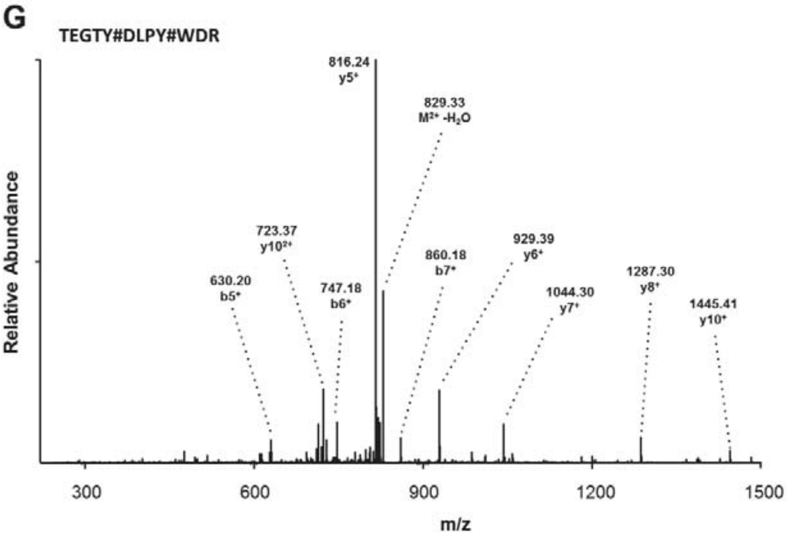
TEGTY#DLPY#WDR
816.24
$y5^{+}$
829.33
M^{2+} -H_2O
723.37
$y10^{2+}$
630.20
$b5^{+}$
747.18
$b6^{+}$
860.18
$b7^{+}$
929.39
$y6^{+}$
1044.30
$y7^{+}$
1287.30
$y8^{+}$
1445.41
$y10^{+}$
Relative Abundance
300
600
900
1200
1500
m/z

H

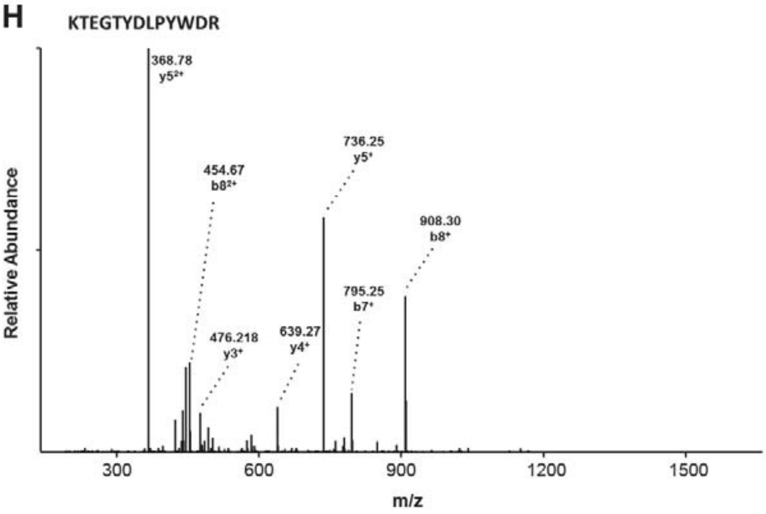
KTEGTYDLPYWDR
368.78
$y5^{2+}$
454.67
$b8^{2+}$
736.25
$y5^{+}$
908.30
$b8^{+}$
795.25
$b7^{+}$
476.218
$y3^{+}$
639.27
$y4^{+}$
Relative Abundance
300
600
900
1200
1500
m/z

I

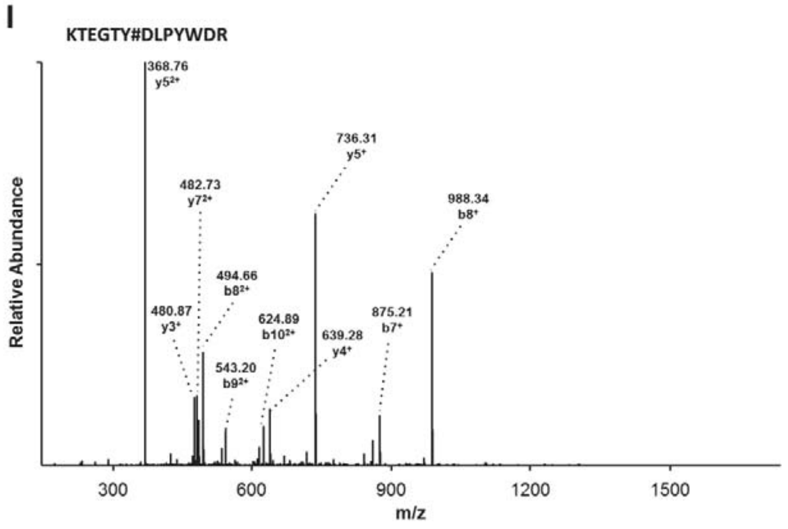
KTEGTY#DLPYWDR
368.76
y5²⁺
736.31
y5⁺
482.73
y7²⁺
988.34
b8⁺
494.66
b8²⁺
480.87
y3⁺
624.89
b10²⁺
875.21
b7⁺
639.28
y4⁺
543.20
b9²⁺
Relative Abundance
300
600
900
1200
1500
m/z

J

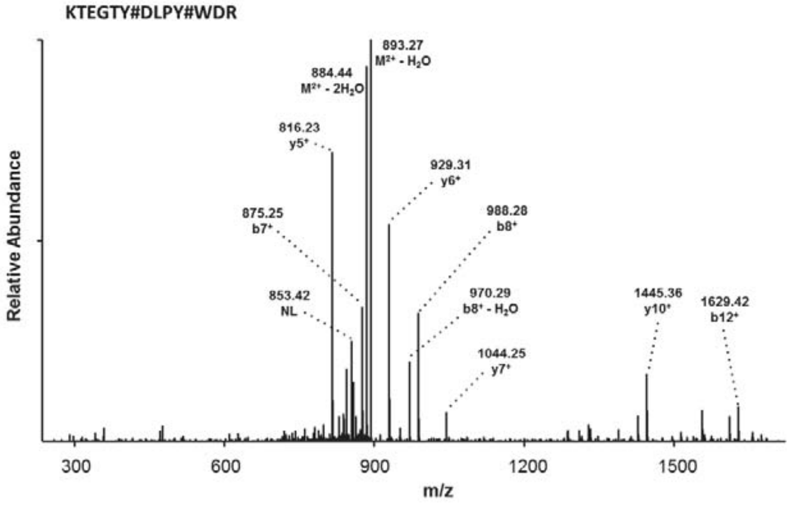
KTEGTY#DLPY#WDR
893.27
M²⁺ - H₂O
884.44
M²⁺ - 2H₂O
816.23
y5⁺
929.31
y6⁺
875.25
b7⁺
988.28
b8⁺
853.42
NL
970.29
b8⁺ - H₂O
1445.36
y10⁺
1629.42
b12⁺
1044.25
y7⁺
Relative Abundance
300
600
900
1200
1500
m/z

K EAGYADLDPYNSPGQEVYHAYAEPLPITGPEYATPIIMDMSGHPTTSVGQPSTSTFK

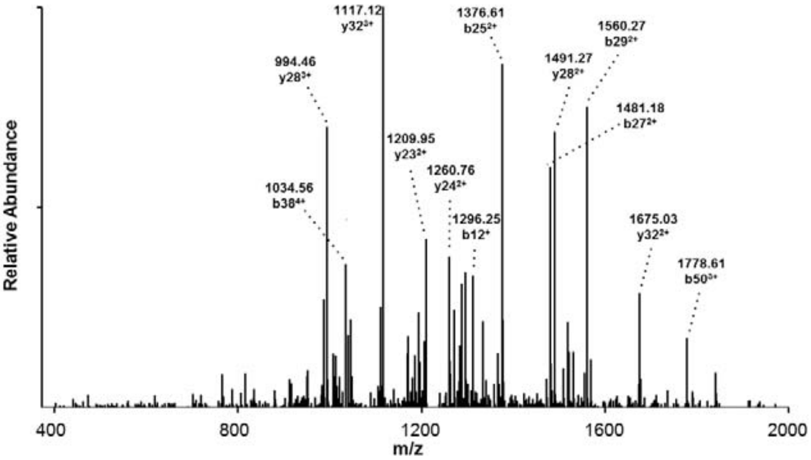

L EAGYADLDPY#NSPGQEVYHAYAEPLPITGPEYATPIIMDMSGHPTTSVGQPSTSTFK

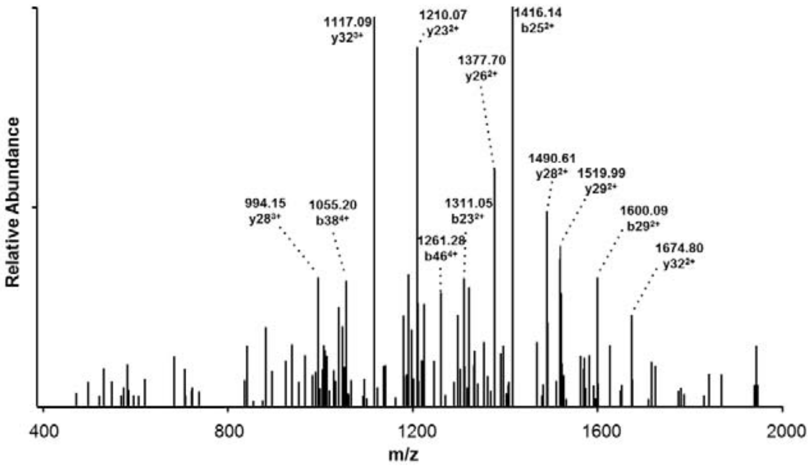

M STFKPEEGKEAGYADLDPYNSPGQEVYHAYAEPLPITGPEYATPIIMDMSGHPTTSVGQPSTSTFK

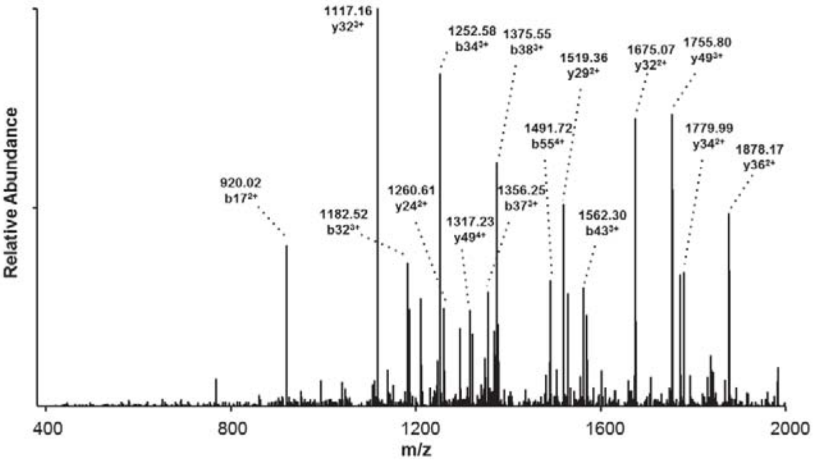

N STFKPEEGKEAGYADLDPYNSPGQEVY#HAYAEPLPITGPEYATPIIMDMSGHPTTSVGQPSTSTFK

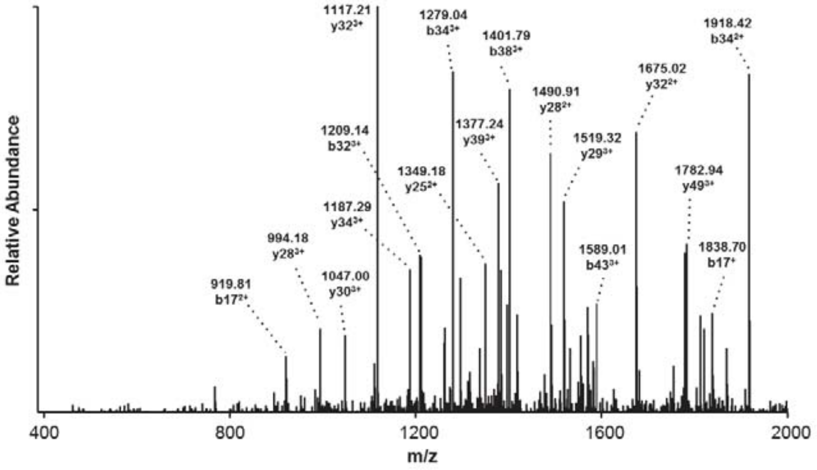

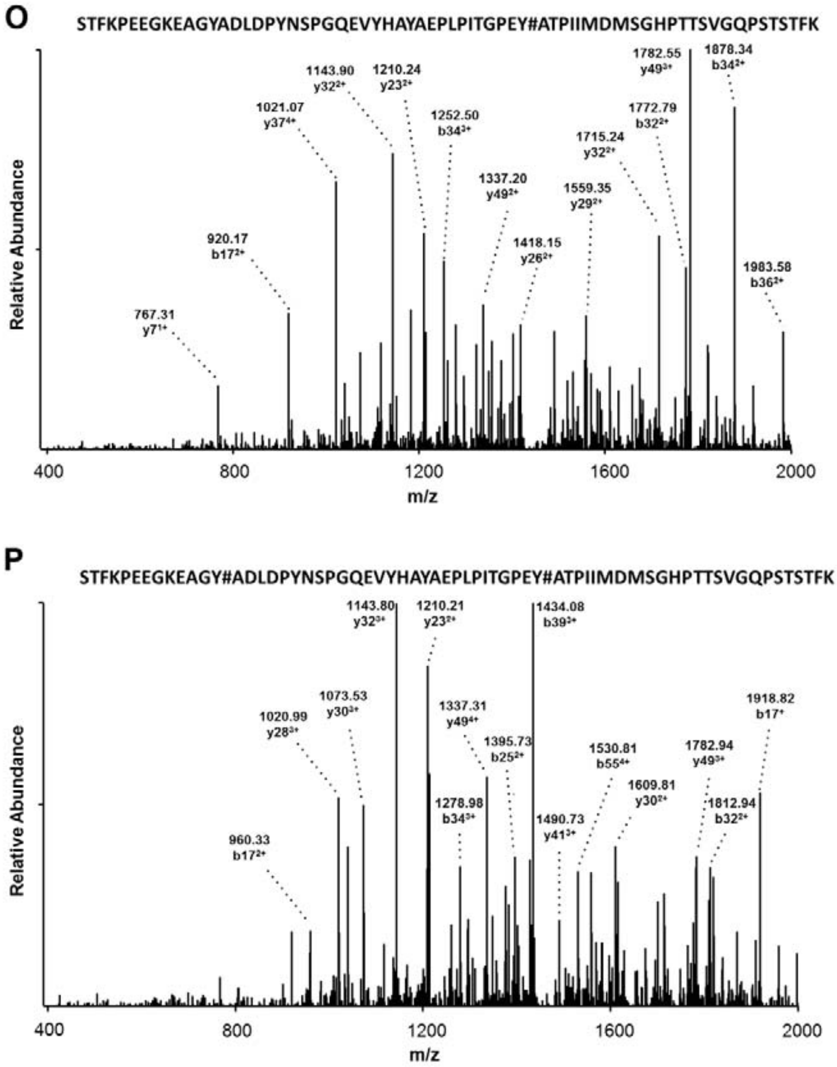
O
STFKPEEGKEAGYADLDPYNSPGQEVYHAYAEPLPITGPEY#ATPIIMDMSGHPTTSVGQPSTSTFK
Relative Abundance
m/z
400
800
1200
1600
2000
767.31 $y7^{1+}$
920.17 $b17^{2+}$
1021.07 $y37^{4+}$
1143.90 $y32^{2+}$
1210.24 $y23^{2+}$
1252.50 $b34^{3+}$
1337.20 $y49^{2+}$
1418.15 $y26^{2+}$
1559.35 $y29^{2+}$
1715.24 $y32^{2+}$
1772.79 $b32^{2+}$
1782.55 $y49^{3+}$
1878.34 $b34^{2+}$
1983.58 $b36^{2+}$
P
STFKPEEGKEAGY#ADLDPYNSPGQEVYHAYAEPLPITGPEY#ATPIIMDMSGHPTTSVGQPSTSTFK
Relative Abundance
m/z
400
800
1200
1600
2000
960.33 $b17^{2+}$
1020.99 $y28^{3+}$
1073.53 $y30^{3+}$
1143.80 $y32^{3+}$
1210.21 $y23^{2+}$
1278.98 $b34^{3+}$
1337.31 $y49^{4+}$
1395.73 $b25^{2+}$
1434.08 $b39^{3+}$
1490.73 $y41^{3+}$
1530.81 $b55^{4+}$
1609.81 $y30^{2+}$
1782.94 $y49^{3+}$
1812.94 $b32^{2+}$
1918.82 $b17^{+}$

Q

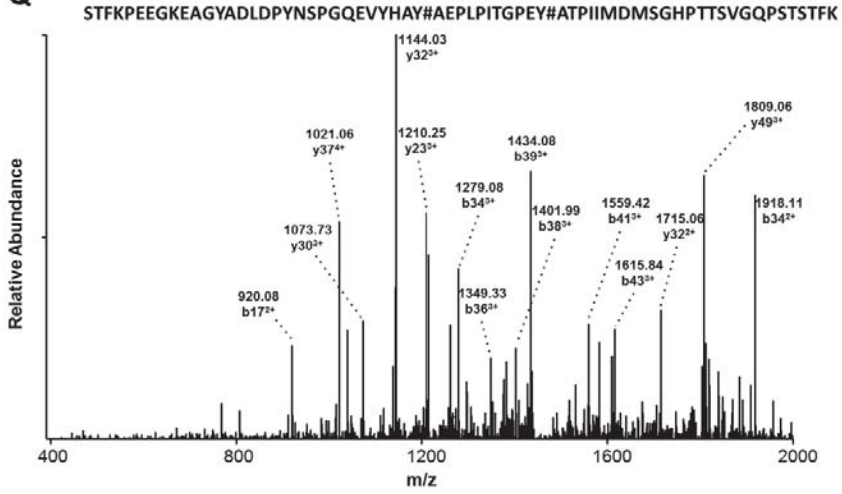
STFKPEEGKEAGYADLDPYNSPGQEVYHAY#AEPLPITGPEY#ATPIIMDMSGHPTTSVGQPSTSTFK
Relative Abundance
m/z
400
800
1200
1600
2000
920.08
b17²⁺
1073.73
y30³⁺
1021.06
y37⁴⁺
1144.03
y32³⁺
1210.25
y23³⁺
1279.08
b34³⁺
1349.33
b36³⁺
1434.08
b39³⁺
1401.99
b38³⁺
1559.42
b41³⁺
1615.84
b43³⁺
1715.06
y32²⁺
1809.06
y49³⁺
1918.11
b34²⁺

Supplementary Table 1. Monoisotopic and average mass to charge (*m/z*) values of (A) DCBLD1 and (B) DCBLD2 unphosphorylated and phosphorylated tryptic peptides harboring differentially regulated tyrosine residues. Amino acid sequences identified with LC-MS/MS are given alongside the charge state and monoisotopic *m/z* values used to quantify peptide intensity, as well as the average *m/z* value, used in targeted scans. Italicized peptides were not targeted, but were identified in data dependent scans.

A

pTyr residue	Peptide	Monoisotopic [m/z]	Average [m/z]	Charge state [z]
Unphos	HEYALPLTHSEPEYATPIVER	818.0763	818.5759	3
pTyr589 or pTyr600	HEYALPLTHSEPEYATPIVER	844.7318	845.2323	3
pTyr589, pTyr600	HEYALPLTHSEPEYATPIVER	871.3872	871.8867	3
Unphos	AHTFSTQSGYR	627.7968	628.1695	2
pTyr621	AHTFSTQSGYR	667.7799	668.1527	2
Unphos	AHTFSTQSGYRVPGPRPTHK	742.0506	742.4962	3
pTyr621	AHTFSTQSGYRVPGPRPTHK	768.706	769.1516	3

B

pTyr residue	Peptide	Monoisotopic [m/z]	Average [m/z]	Charge state [z]
Unphos	TEGTYDLPYWDR	758.3412	758.8067	2
pTyr565	TEGTYDLPYWDR	798.3244	798.7899	2
pTyr565, pTyr569	*TEGTYDLPYWDR*	*838.3075*	*838.773*	*2*
Unphos	KTEGTYDLPYWDR	548.5949	548.9316	3
pTyr565	KTEGTYDLPYWDR	575.2503	575.587	3
pTyr565, pTyr569	*KTEGTYDLPYWDR*	*902.355*	*902.86*	*2*
Unphos	EVTTVLQADSAEYAQPLVGGIVGTLHQR	984.8524	985.4413	3
pTyr621	EVTTVLQADSAEYAQPLVGGIVGTLHQR	1011.5078	1012.097	3
Unphos	*EAGYADLDPYNSPGQEVYHAYAEPLPITGPEYATPIIMDMSGHPTTSVGQPSTSTFK*	*1220.5711*	*1221.34*	*5*
pTyr 655	*EAGYADLDPYNSPGQEVYHAYAEPLPITGPEYATPIIMDMSGHPTTSVGQPSTSTFK*	*1236.3628*	*1237.1344*	*5*
Unphos	*STFKPEEGKEAGYADLDPYNSPGQEVYHAYAEPLPITGPEYATPIIMDMSGHPTTSVGQPSTSTFK*	*1421.2706*	*1422.1608*	*5*
Singly pTyr	*STFKPEEGKEAGYADLDPYNSPGQEVYHAYAEPLPITGPEYATPIIMDMSGHPTTSVGQPSTSTFK*	*1437.0623*	*1437.9552*	*5*
Doubly pTyr	*STFKPEEGKEAGYADLDPYNSPGQEVYHAYAEPLPITGPEYATPIIMDMSGHPTTSVGQPSTSTFK*	*1452.854*	*1453.7496*	*5*
Unphos	ATGNQPPPLVGTYNTLLSR	1000.034	1000.6344	2
pTyr715	ATGNQPPPLVGTYNTLLSR	1040.0172	1040.6175	2
Unphos	AGKPGLPAPDELVYQVPQSTQEVSGAGR	951.1577	951.7251	3
pTyr750	AGKPGLPAPDELVYQVPQSTQEVSGAGR	977.8132	978.3805	3

Supplementary Table 2. Monoisotopic and average *m/z* values of tryptic peptides harboring targeted tyrosine and phosphotyrosine residues for (A) DCBLD2 SL peptides, (B) DCBLD1 heavy-labeled SILAC peptides, and (C) DCBLD2 heavy-labeled SILAC peptides. Underlined residues possess all ^{13}C and ^{15}N atoms, resulting in added masses of 7.0172 *m/z* for Leu, 6.0138 *m/z* for Val, 10.0083 *m/z* for Arg, and 8.0142 *m/z* for Lys.

A

**pTyr residue**	**Peptide**	**Monoisotopic** [m/z]	**Average** [m/z]	**Charge state** [z]
Unphos	KTEGTYDLPYWDR	550.934	551.2707	3
pTyr565	KTEGTYDLPYWDR	577.5894	577.9261	3
Unphos	ATGNQPPPLVGTYNTLLSR	1003.5426	1004.143	2
pTyr715	ATGNQPPPLVGTYNTLLSR	1043.5258	1044.1261	2
Unphos	AGKPGLPAPDELVYQVPQSTQEVSGAGR	953.1623	953.7297	3
pTyr750	AGKPGLPAPDELVYQVPQSTQEVSGAGR	979.8178	980.3851	3

B

**pTyr residue**	**Peptide**	**Monoisotopic** [m/z]	**Average** [m/z]	**Charge state** [z]
Unphos	HEYALPLTHSEPEYATPIVER	821.4124	821.912	3
pTyr589 or pTyr600	HEYALPLTHSEPEYATPIVER	848.0679	848.5674	3
pTyr589, pTyr600	HEYALPLTHSEPEYATPIVER	874.7233	875.2228	3
Unphos	AHTFSTQSGYR	632.8009	633.1737	2
pTyr621	AHTFSTQSGYRVPGPRPTHK	778.0496	778.4952	3

C

**(p)Tyr residue**	**Peptide**	**Monoisotopic** [m/z]	**Average** [m/z]	**Charge state** [z]
Unphos	TEGTYDLPYWDR	763.3454	763.8109	2
pTyr565	TEGTYDLPYWDR	843.3117	843.7772	2
Unphos	KTEGTYDLPYWDR	554.6024	554.9391	3
pTyr565	KTEGTYDLPYWDR	607.9133	608.25	3
Unphos	EVTTVLQADSAEYAQPLVGGIVGTLHQR	988.1885	988.7774	3
pTyr621	EVTTVLQADSAEYAQPLVGGIVGTLHQR	1014.8439	1015.4328	3
Unphos	ATGNQPPPLVGTYNTLLSR	1005.0382	1005.6385	2
pTyr715	ATGNQPPPLVGTYNTLLSR	1045.0213	1045.6217	2
Unphos	AGKPGLPAPDELVYQVPQSTQEVSGAGR	957.1652	957.7326	3
pTyr750	AGKPGLPAPDELVYQVPQSTQEVSGAGR	983.8207	984.388	3

Supplementary Table 3. Monoisotopic and average *m/z* values of tryptic peptides of (A) DCBLD1 and (B) DCBLD2 reference peptides used in SILAC, SL peptide and LF quantification. Light and heavy masses are given with charge state and the quantification method that employed each reference. The italicized peptide was targeted in SL quantification experiments. All remaining peptides were identified in data dependent scans. Underlined residues possess all ^{13}C and ^{15}N atoms, resulting in added masses of 6.0138 *m/z* for Val, 10.0083 *m/z* for Arg, and 8.0142 *m/z* for Lys.

A

**pTyr residue**	**Peptide**	**Monoisotopic** [m/z]	**Average** [m/z]	**Charge state** [z]	**Labeling (method)**
114-124	LNSNEVTVLFK	632.3509	632.7355	2	Native (LF, SILAC)
114-124	LNSNEVTVLFK	636.358	636.7426	2	Heavy (SILAC)
153-160	GSHYFEEK	498.7248	499.0293	2	Native (LF, SILAC)
153-160	GSHYFEEK	502.7319	503.0364	2	Heavy (SILAC)
171-181	DIAGDISGNTK	545.7724	546.0849	2	Native (LF, SILAC)
171-181	DIAGDISGNTK	549.7795	550.092	2	Heavy (SILAC)

B

**(p)Tyr residue**	**Peptide**	**Monoisotopic** [m/z]	**Average** [m/z]	**Charge state** [z]	**Labeling (method)**
133-141	IYNGIGVSR	489.772	490.0653	2	Native (LF, SILAC)
133-141	IYNGIGVSR	494.7762	495.0695	2	Heavy (SILAC)
179-189	*GFLASYSVIDK*	*600.319*	*600.6918*	*2*	*Native (SL)*
179-189	GFLASYSVIDK	603.3259	603.6987	2	Heavy (SL)
418-427	NNFLPPIIAR	577.8377	578.1961	2	Native (LF, SILAC)
418-427	NNFLPPIIAR	582.8419	583.2002	2	Heavy (SILAC)
489-496	FTQPLQPR	493.7746	494.0766	2	Native (LF, SILAC)
489-496	FTQPLQPR	498.7787	498.0808	2	Heavy (SILAC)

Supplementary Table 4. SILAC heavy-to-light ratios (fold change) of DCBLD1 and DCBLD2 unphosphorylated peptides harboring tyrosines differentially regulated by SFKs and Abl. Heavy conditions possessed either a co-expressed kinase (paired with a light unstimulated condition), or H_2O_2 stimulation alone (paired with a light inhibitor treatment following H_2O_2 stimulation). The peptide sequences and the number of the regulated tyrosine residues (human numbering) are indicated on the left.

		**Heavy condition** (Light = unstimulated)				**Light condition** (Heavy = H_2O_2)	
	**Peptide**	**H_2O_2**	**Fyn**	**c-Abl**	**Fyn & c-Abl**	**Src-1 H_2O_2**	**STI571 H_2O_2**
**DCBLD1**	**HEYALPLTHSEPEYATPIVER** Tyr 589 Tyr600	0.75	0.78	0.31	0.45	0.75	0.73
	**AHTFSTQSGYR** Tyr621	1.18	1.13	0.54	0.76	1.09	1.09
**DCBLD2**	**TEGTYDLPYWDR** Tyr565	0.72	0.50	0.92	0.49	0.82	0.49
	**EVTTVLQADSAEYAQPLVGGIVGTLHQR** Tyr621	0.60	0.70	0.42	0.41	0.74	0.64
	**ATGNQPPPLVGTYNTLLSR** Tyr715	0.76	0.16	0.53	0.63	0.83	0.73
	**AGKPGLPAPDELVYQVPQSTQEVSGAGR** Tyr750	0.46	0.75	0.20	0.19	0.60	0.46

Supplementary Table 5. Statistical analyses of DCBLD2 phosphopeptide quantificaiton. DCBLD2 peptides harboring quantified Tyr phosphorylation sites were subjected to a one-way ANOVA and post-hoc Tukey HSD to identify statistical differences in phosphorylation of each peptide across experimental conditions. Conditions with statistically significant differences (P = 0.05) are tabulated for (A) Tyr565 and (B) Tyr715 and Tyr750. K = kinase overexpressed group, I = inhibitor treatment group. K/TEGTYDLPYWDR denotes summed intensities of KTEGTYDLPYWDR and TEGTYDLPYWDR peptides. "Y#" denotes phosphorylated tyrosine.

A

			one-way ANOVA (0.05)	*Tukey All Pairs (0.05)*		
**Peptide, (p)Tyr residue**	**Method**	**Group**	***P*-value**	**Condition 1**	**Condition 2**	***P*-value**
**K/TEGTY#DLPYWDR, pTyr565**	LF	K	3.00E-04	Fyn & c-Abl	*Unstimulated*	2.00E-04
				H_2O_2	*Unstimulated*	5.50E-03
				Fyn & c-Abl	*c-Abl*	6.10E-03
				Fyn	*Unstimulated*	1.06E-02
**KTEGTY#DLPYWDR, pTyr565**	SL	K	3.41E-02	Fyn	*Unstimulated*	3.88E-02
**KTEGTY#DLPYWDR, pTyr565**	LF	K	4.30E-03	Fyn & c-Abl	*Unstimulated*	5.20E-03
				Fyn & c-Abl	*c-Abl*	1.72E-02
				H_2O_2	*Unstimulated*	3.18E-02
**TEGTY#DLPYWDR, pTyr565**	LF	K	4.00E-04	Fyn & c-Abl	*Unstimulated*	2.00E-04
				Fyn & c-Abl	*c-Abl*	9.40E-03
				H_2O_2	*Unstimulated*	9.40E-03
				Fyn	*Unstimulated*	1.21E-02
**KTEGTY#DLPYWDR, Tyr565**	SL	K	3.41E-02	Fyn	*Unstimulated*	3.88E-02
**K/TEGTY#DLPYWDR, pTyr565** summed intensities	LF	I	1.20E-03	H_2O_2	*Unstimulated*	3.90E-03
				H_2O_2	*Fyn & c-Abl*	4.40E-03
				c-Abl	*Unstimulated*	1.18E-02
				c-Abl	*Fyn & c-Abl*	1.34E-02
				H_2O_2	*Fyn*	3.83E-02
**KTEGTY#DLPYWDR, pTyr565**	SL	I	2.69E-02	c-Abl	*Unstimulated*	4.39E-02
**TEGTY#DLPYWDR, pTyr565**	LF	I	<1.00E-04	H_2O_2	*Unstimulated*	<1.00E-04
				H_2O_2	*Fyn & c-Abl*	1.00E-04
				H_2O_2	*Fyn*	1.20E-03
				H_2O_2	*c-Abl*	3.60E-03
				c-Abl	*Unstimulated*	7.35E-02

B

Peptide and/or (p)Tyr residue	Method	Group	*one-way ANOVA (0.05)* *P*-value	*Tukey All Pairs (0.05)* Condition 1	Condition 2	*P*-value
**ATGNQPPPLVGTY#NTLLSR pTyr715**	SL	K	1.13E-02	Fyn & c-Abl	*Unstimulated*	1.29E-02
				c-Abl	*Unstimulated*	3.10E-02
**ATGNQPPPLVGTY#NTLLSR pTyr715**	LF	K	2.17E-02	c-Abl	*Unstimulated*	2.29E-02
				Fyn & c-Abl	*Unstimulated*	3.77E-02
**ATGNQPPPLVGTY#NTLLSR pTyr715**	SL	I	2.51E-02	H_2O_2	*Unstimulated*	2.38E-02
				H_2O_2	*Fyn & c-Abl*	3.55E-02
**ATGNQPPPLVGTY#NTLLSR pTyr715**	LF	I	<1.00E-04	H_2O_2	*Unstimulated*	<1.00E-04
				H_2O_2	*Fyn & c-Abl*	<1.00E-04
				H_2O_2	*Fyn*	<1.00E-04
				H_2O_2	*c-Abl*	4.00E-04
				c-Abl	*Unstimulated*	2.68E-02
				c-Abl	*Fyn & c-Abl*	3.94E-02
**AGKPGLPAPDELVY#QVPQSTQEVSGAGR pTyr750**	SL	K		Fyn & c-Abl	*Unstimulated*	7.00E-04
				H_2O_2	*Unstimulated*	1.30E-03
				c-Abl	*Unstimulated*	2.80E-03
				Fyn & c-Abl l	*Fyn*	1.31E-02
				H_2O_2	*Fyn*	2.77E-02
**AGKPGLPAPDELVY#QVPQSTQEVSGAGR pTyr750**	LF	K	3.30E-03	Fyn & c-Abl	*Unstimulated*	3.40E-03
				c-Abl	*Unstimulated*	1.46E-02
				H_2O_2	*Unstimulated*	1.66E-02
**AGKPGLPAPDELVY#QVPQSTQEVSGAGR Tyr750**	LF	K	4.50E-03	c-Abl	*Unstimulated*	7.90E-03
				H_2O_2	*Unstimulated*	2.28E-02
				c-Abl	*Fyn*	3.17E-02
				Fyn & c-Abl	*Unstimulated*	4.72E-02

Supplementary Table 6. Unquantified DCBLD2 tryptic peptides identified via LC-MS/MS. “Y#” denotes site of tyrosine phosphorylation. Annotated fragmentation spectra can be found in Supplemental Figure 6.

	Tyr residue	Peptide
Inducible by H_2O_2	Tyr565, Tyr569	K.TEGTY#DLPY#WDR
	Tyr 655	EAGYADLDPY#NSPGQEVYHAYAEPLPITGPEYATPIIMDMSGHPTTSVGQPSTSTFK
	Tyr649, Tyr677	STFKPEEGKEAGY#ADLDPYNSPGQEVYHAYAEPLPITGPEY#ATPIIMDMSGHPTTSVGQPSTSTFK
	Tyr663	STFKPEEGKEAGYADLDPYNSPGQEVY#HAYAEPLPITGPEYATPIIMDMSGHPTTSVGQPSTSTFK
	Tyr677	STFKPEEGKEAGYADLDPYNSPGQEVYHAYAEPLPITGPEY#ATPIIMDMSGHPTTSVGQPSTSTFK
	Tyr666, Tyr677	STFKPEEGKEAGYADLDPYNSPGQEVYHAY#AEPLPITGPEY#ATPIIMDMSGHPTTSVGQPSTSTFK
